# Inflammation-driven immune reprogramming in sepsis: from cytokine storm to immunoparalysis

**DOI:** 10.3389/fimmu.2026.1887033

**Published:** 2026-07-09

**Authors:** Sha Zhu, Ji He

**Affiliations:** 1 Department of Adult Critical Care Medicine, West China Second University Hospital, Sichuan University, Chengdu, China; 2 Key Laboratory of Birth Defects and Related Diseases of Women and Children (Sichuan University), Ministry of Education, Chengdu, China

**Keywords:** clinical trial design, dynamic immune monitoring, endotypes, immune reprogramming, immune trajectories, immunoparalysis, precision immunotherapy, sepsis

## Abstract

Sepsis remains a leading cause of preventable death, partly because its rapidly evolving and heterogeneous biology has often been treated as a uniform inflammatory syndrome in therapeutic development and clinical trials. This narrative review synthesizes recent advances in human immune profiling to reframe sepsis as an inflammation-driven immune reprogramming disorder characterized by time-resolved trajectories and biologically defined subtypes, in which hyperinflammation and low-response programs can arise in parallel and shift across compartments. We summarize core mechanisms linking early innate sensing, endothelial injury, coagulation–inflammation coupling, and cytokine network “instruction” to later immune paralysis, lymphocyte loss, functional exhaustion, and the chronic critical illness phenotype. We then translate these concepts into a practical interpretive framework for dynamic immune monitoring, emphasizing trends and composite signals over single biomarkers, and highlighting how misalignment of patient state, therapeutic direction, and timing helps explain past trial failures. Finally, we outline implications for next-generation interventions and study designs, arguing for bidirectional, time-stamped immunomodulation guided by repeated phenotyping, enriched enrollment, and endpoints that capture secondary infections, organ recovery trajectories, and longer-term functional outcomes.

## 1 Introduction

### 1.1 Background and clinical–scientific significance

Sepsis is defined as life-threatening organ dysfunction caused by a dysregulated host response to infection, and this definition is operationalized in the Surviving Sepsis Campaign guidelines that shape current intensive care unit (ICU) practice worldwide (1). Despite major gains in early recognition, antimicrobial therapy, source control, and protocolized supportive care, sepsis remains a leading cause of death and a major driver of long-term disability among survivors (2). These realities underscore that sepsis is not merely a failure of pathogen clearance. Rather, it is an inflammation-driven syndrome in which immune programs are rewired in ways that both mediate acute tissue injury and undermine later protective responses (2, 3). Clinically, this immune reprogramming helps explain why some patients deteriorate despite microbiologic control, why secondary infections and viral reactivation are common, and why many survivors experience prolonged weakness, cognitive decline, and recurrent hospitalizations (2, 3). Accordingly, contemporary immunobiology reframes sepsis along a dynamic trajectory “from cytokine storm to immunoparalysis,” recognizing that excessive inflammatory signaling and compensatory immune suppression are mechanistically linked and often temporally overlapping (3). This review adopts that framework to connect molecular inflammation to immune-cell state shifts and clinically meaningful outcomes.

### 1.2 Core concepts and roadmap of this review

“Hyperinflammation” denotes the early (or dominant) inflammatory component of sepsis, marked by rapid innate immune sensing, escalating cytokine cascades, endothelial activation, and feed-forward parenchymal injury. Systems-level analyses further suggest that cytokine responses form hierarchical modules, grounding the concept of a “cytokine storm” in definable network logic (4). “Immunoparalysis” refers to a counter-regulatory state characterized by impaired antigen presentation, reduced effector cytokine production, lymphocyte apoptosis, and diminished pathogen clearance—features that correlate with secondary infection risk and late mortality (2, 3). “Immune exhaustion” describes a related but more specific, durable hyporesponsive program driven by sustained inflammatory stress, inhibitory checkpoint signaling, and metabolic insufficiency, particularly within T cell and natural killer (NK) cell compartments (3). Persistent inflammation, immunosuppression, and catabolism syndrome (PICS) extends these concepts to chronic critical illness, capturing patients who fail to return to immune homeostasis and instead remain locked in a maladaptive inflammatory–suppressive equilibrium (5).

The roadmap of this review follows a causal chain: inflammatory signaling and mediator networks → immune cell reprogramming in phenotype, metabolism, and epigenetic memory → downstream consequences including secondary infection, organ injury, and long-term sequelae. Single-cell profiling supports this logic by showing that sepsis imprints structured, lineage-spanning state changes on circulating immune cells—such as suppressive myeloid programs and contracted lymphocyte compartments—consistent with coordinated reprogramming rather than nonspecific immune “collapse” (6–9). Throughout, we emphasize that reprogramming implies state remodeling and functional biasing, not a simple linear slide into immunodeficiency.

To standardize terminology, **Table 1** summarizes the key definitions used throughout this review, alongside typical biomarkers and common points of confusion (3, 5, 10–12).

**Table 1:** Key definitions & concept map.

Term	Operational definition (as used here)	Key features	Typical indicators/common confusions
Hyperinflammation	Early or dominant inflammatory component of sepsis driven by innate sensing, cytokine cascades, endothelial and coagulation activation.	Can be partly host-protective but becomes injurious when excessive; often co-exists with emerging low-response programs.	Indicators: high inflammatory mediators, endothelial/platelet activation, tissue-injury markers. Confusions: treated as an isolated “phase” that must precede suppression or as uniformly harmful.
Immunoparalysis	Time-evolving low-responsiveness of the immune system with impaired antigen presentation, cytokine production, and pathogen clearance.	Dynamic, compartment- and time-dependent; linked to secondary infection and late mortality, not a single lab cut-off.	Indicators: low monocyte HLA-DR, lymphopenia, blunted ex vivo responses. Confusions: used interchangeably with CARS; assumed to be purely “late” or defined by one biomarker.
Immune exhaustion	Longer-lived hyporesponsive program, especially in T/NK cells, driven by sustained antigenic and checkpoint signaling.	Considered a component of immunoparalysis, not a separate syndrome; mainly affects adaptive and NK compartments.	Indicators: checkpoint upregulation with reduced effector function/proliferation. Confusions: mixed up with simple depletion/apoptosis or immunosenescence; treated as a static marker rather than an active circuit.
PICS	Persistent Inflammation–Immunosuppression and Catabolism Syndrome: failure to return to immune and metabolic homeostasis after sepsis.	Triad of low-grade inflammation, sustained immune suppression, and catabolic frailty; typical of prolonged ICU stays.	Indicators: chronic inflammatory markers, low HLA-DR/functional defects, muscle wasting and poor recovery. Confusions: equated with any long ICU stay or generic “post-sepsis syndrome,” ignoring the catabolic component.
Endotype	Biologically defined patient subgroup characterized by shared immune circuits or mediator patterns beyond clinical phenotype or severity scores.	Can be storm-dominant, suppression-dominant, or mixed; may evolve over time but captures mechanistic differences.	Indicators: reproducible transcriptomic/cellular clusters or composite signatures. Confusions: endotype vs phenotype; assumed to be fixed rather than revisited with serial profiling.
Trajectory	Time-resolved immune course an individual patient follows over hours to weeks.	Emphasizes serial, time-stamped changes; hyperinflammation and low-responsiveness may appear in parallel; requires re-typing.	Indicators: longitudinal trends in biomarkers and clinical events (e.g., infection burden, organ-failure slopes). Confusions: trajectory (within-patient evolution) vs endotype (between-patient subgroup); oversimplified as a universal SIRS→CARS switch.

Terms are defined operationally for use in this review. “Endotype” denotes a biologically or mechanistically defined subgroup (between-patient), whereas “trajectory” denotes within-patient, time-resolved evolution; these constructs can overlap and should be interpreted in a time-stamped, compartment-aware manner. The “typical indicators/common confusions” column highlights pragmatic readouts and frequent interpretive pitfalls rather than diagnostic cutoffs. PICS, persistent inflammation–immunosuppression and catabolism syndrome; mHLA-DR, monocyte human leukocyte antigen–DR; CARS, compensatory anti-inflammatory response syndrome; ICU, intensive care unit. “Endotype” denotes a mechanistic subgroup, whereas “trajectory” denotes within-patient, time-resolved evolution; these constructs can overlap and should be interpreted in a time-stamped manner.

### 1.3 Recent advances and review objectives

Over the past five years, sepsis immunology has been transformed by single-cell and spatially informed multi-omic approaches that resolve immune trajectories, cell–cell communication, and stable suppressive programs *in vivo* (6, 7, 13). In parallel, cross-cohort host-transcriptomic research has converged on transferable immune endotypes, consensus dysregulation frameworks, and quantitative immune-dysfunction scores, offering a shared language for heterogeneity and a platform for precision immunomodulation (14–17). These advances have renewed interest in bedside immunomonitoring and endotype-guided therapy, as reflected by emerging protocolized trials that stratify patients according to immune status before testing targeted interventions (18).

Against this background, this review has three objectives. First, it synthesizes how early inflammatory signaling, endothelial injury, coagulation–inflammation coupling, and cytokine-network instruction drive immune-cell fate remodeling in sepsis. Second, it connects hyperinflammation, immunoparalysis, immune exhaustion, and persistent inflammation–immunosuppression and catabolism syndrome within a unified trajectory- and endotype-based framework. Third, it discusses how dynamic immune monitoring may inform precision immunomodulation, patient enrichment, therapeutic timing, and next-generation trial design.

## 2 Methods

This narrative review used a targeted, question-driven literature search rather than a systematic-review or meta-analysis protocol. We searched MEDLINE (PubMed), Embase (Embase.com), and Scopus for literature published from 1 January 2020 to 31 December 2025. Foundational primary studies published before 2020 were additionally cited when they provided original evidence for established sepsis endotypes, immune-monitoring biomarkers, or immunomodulatory interventions. Two complementary search strategies were used. Search A focused on mechanistic and immune-phenotyping evidence related to sepsis immune reprogramming, immune trajectories, endotypes, single-cell profiling, transcriptomics, and multi-omic studies. Search B focused on translational evidence related to sepsis-associated immunoparalysis or immune suppression, immune monitoring, biomarkers, immunomodulatory interventions, and trial design.

English-language human studies were prioritized, and database-specific filters for Humans and English were applied where available. We included international guidelines, major review articles, representative human cohort studies, translational immune-profiling studies, and contemporary syntheses that directly informed the conceptual framework of inflammation-driven immune reprogramming in sepsis. Studies were excluded when they did not address sepsis or critical illness, lacked relevance to immune reprogramming or immune monitoring, or focused solely on non-human experimental models without clear translational relevance.

Records were exported to EndNote 21, with duplicates removed first within the combined Search A and Search B outputs for each database and then across databases after merging. Titles, abstracts, and full texts were screened for relevance to the predefined review questions. Evidence was narratively synthesized by mechanistic domain, immune trajectory, biomarker category, and therapeutic implication. Database-specific search strings and record accounting are provided in the **Supplementary Methods**. Because this review synthesized published literature only, ethics approval was not required.

## 3 Conceptual framework: sepsis as an immune reprogramming syndrome

### 3.1 Evolution from “single-phase inflammation” to biphasic and mixed/parallel immune-response models

Sepsis was historically viewed as a prototypical “single-phase” inflammatory disorder in which an early systemic inflammatory response syndrome (SIRS) dominated pathogenesis and dictated outcomes. The SIRS→compensatory anti-inflammatory response syndrome biphasic paradigm emerged when clinicians recognized that many patients who survived the initial shock later developed opportunistic infections, viral reactivation, and prolonged ICU courses. Conceptually, compensatory anti-inflammatory response syndrome framed these later events as a compensatory counter-reaction to the early storm, providing an intuitively time-ordered narrative that aligned with bedside observations and guided decades of anti-inflammatory trial design (10, 19). The biphasic model was also compelling because it offered a mechanistic bridge between early organ injury and later immune incompetence and suggested a clear therapeutic sequence: suppress inflammation early, then restore immunity later (3, 10, 19).

However, evidence from the past five years indicates that this linear switch is an oversimplification. Contemporary immune profiling and transcriptomic cohort studies show that pro-inflammatory activation and immunosuppressive circuitry can coexist from the earliest hours of sepsis, rather than waiting for a late “phase change” (6, 9, 14, 15). High cytokine and alarmin signaling is often accompanied by early monocyte deactivation, lymphocyte dysfunction/apoptosis, checkpoint upregulation, and expansion of suppressive myeloid programs, yielding mixed/parallel immune states that are detectable at presentation (6, 7, 19–21). Longitudinal analyses further support trajectory-based models: patients follow heterogeneous immune courses shaped by baseline biology and ongoing insults, with some remaining hyperinflammatory, others rapidly developing immunoparalysis, and many exhibiting overlapping features over time (11, 14, 21, 22). This shift reframes sepsis as dynamic immune dysregulation—variable in timing, compartment, and host endotype—rather than a predictable, population-level biphasic sequence (11, 22).

**Figure 1:**
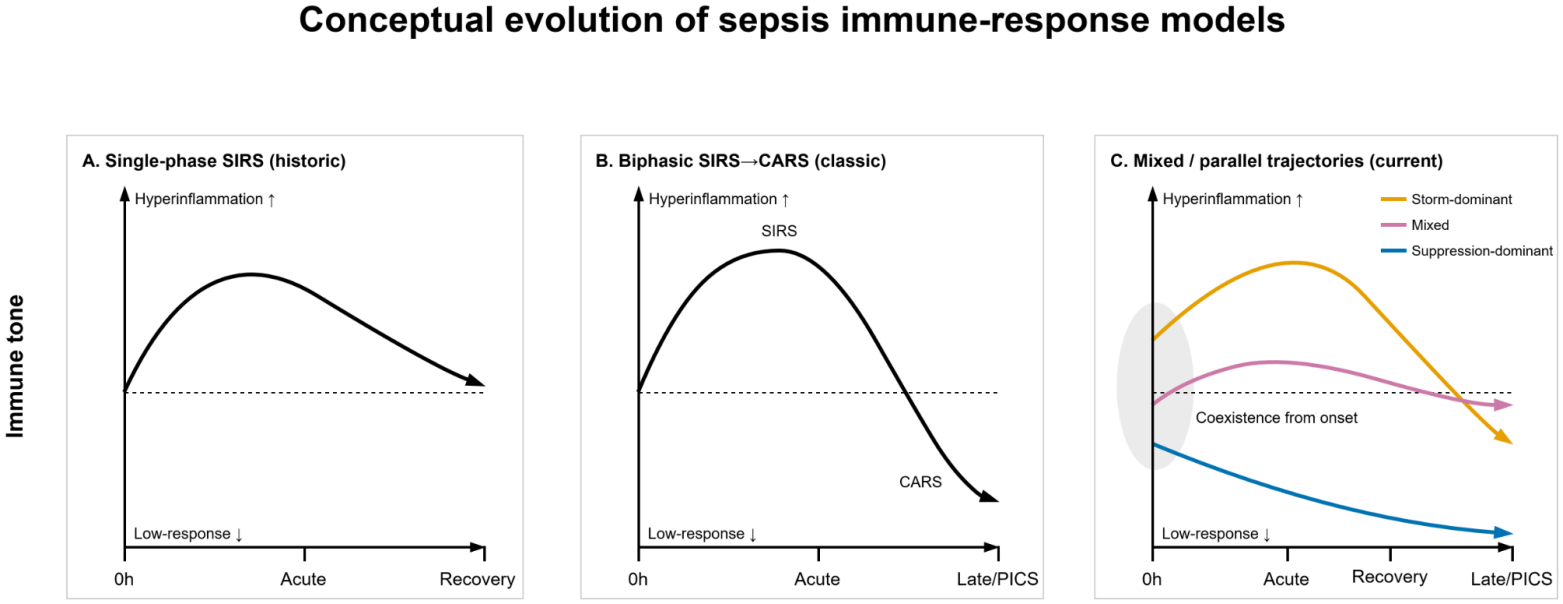
Conceptual evolution of sepsis immune-response models. Conceptual schematic (not to scale) illustrating how views of the sepsis immune response have shifted over time. **(A)** Early single-phase model emphasizing a transient SIRS with return toward baseline once hyperinflammation resolves. **(B)** Classic biphasic SIRS→CARS paradigm proposing a sequential shift from an initial hyperinflammatory phase to a later CARS and net immunosuppression. **(C)** Contemporary mixed/parallel framework in which pro- and anti-inflammatory programs coexist from onset and diverge into heterogeneous trajectories, exemplified by storm-dominant, mixed, and suppression-dominant courses. The dashed horizontal line indicates the approximate homeostatic baseline of immune tone, and the grey shaded area highlights early coexistence of activation and suppression. SIRS, systemic inflammatory response syndrome; CARS, compensatory anti-inflammatory response syndrome; PICS, persistent inflammation–immunosuppression–catabolism syndrome.

**Figure 1** illustrates this conceptual evolution from a historic single-phase SIRS model, through the classic SIRS→compensatory anti-inflammatory response syndrome paradigm, to contemporary mixed/parallel immune-response trajectories.

### 3.2 Definition and multi-level layers of immune reprogramming

Building on the clinical definition of sepsis as life-threatening organ dysfunction driven by a dysregulated host response to infection (1), “immune reprogramming” here refers to quantitative, qualitative, and temporal remodeling of immunity induced by infectious and inflammatory stress—not a unidirectional slide into immune weakness (7, 19, 21). Quantitatively, sepsis reshapes immune composition through lymphocyte apoptosis, emergency myelopoiesis, altered trafficking, and tissue sequestration, producing characteristic lymphopenia alongside expansion of innate myeloid populations (7, 19, 23). Qualitatively, surviving cells adopt rebased phenotypes and functions: monocytes and dendritic cells show reduced human leukocyte antigen DR (HLA-DR) expression and tolerogenic signaling; neutrophils and immature granulocytes acquire suppressive or tissue-injurious programs; and adaptive cells display exhaustion-like features with diminished cytokine output and cytotoxicity (7, 19, 21, 23). Temporally, these changes evolve over hours to weeks, leaving persistent functional “set points” that shape subsequent responses (11, 21, 23).

Reprogramming operates across interconnected biological layers. At the cellular level, it reflects coordinated state shifts in innate and adaptive arms, including differentiation, activation thresholds, and effector repertoires (7, 19, 23). At the molecular level, transcriptomic signatures demonstrate stable pathway rewiring toward checkpoint-rich, stress-tolerant, and metabolically constrained states; immunometabolic remodeling and epigenetic changes provide inflammatory memory that biases later antimicrobial and inflammatory outputs (14, 19, 21). At the system and organ microenvironment level, compartment-specific immune states arise within lung, gut, kidney, and vascular niches, shaped by barrier failure, endothelial activation, damage-associated molecular pattern (DAMP) and pathogen-associated molecular pattern (PAMP) flux, and organ crosstalk (11, 19, 21). Such multilevel reprogramming links acute survival to later vulnerability, helping explain secondary infection risk, persistent inflammation–catabolism, and chronic critical illness phenotypes after sepsis (5, 21, 23).

### 3.3 Balanced summary of the major schools/debates

Two interpretive schools continue to shape the field. The storm-centric view holds that early uncontrolled inflammation is the primary driver of endothelial injury, microvascular collapse, and organ dysfunction, with later immunosuppression largely a downstream consequence of the initial cytokine-mediated insult (3, 10, 19). This perspective emphasizes the causal weight of hyperinflammatory signaling and tissue damage in setting the trajectory of critical illness. In contrast, the immunosuppression-centric view argues that immunoparalysis and immune exhaustion—manifested by impaired antigen presentation, checkpoint dominance, dysfunctional lymphocytes, and reduced pathogen control—are decisive for late mortality and secondary infection and may emerge early enough to warrant prompt correction (19–21, 23).

A reconciliatory synthesis is increasingly supported by endotype- and trajectory-based frameworks. Multi-cohort RNA sequencing integration identifies molecular endotypes that differ in inflammatory–suppressive balance, kinetics, and outcomes, underscoring that sepsis is biologically heterogeneous rather than phase-uniform (22). The 2025 consensus dysregulation framework similarly emphasizes time- and compartment-dependent immune trajectories in which hyperinflammation, immunoparalysis, and mixed states variably overlap across patients and organs (16). Therefore, neither single-dominance view is sufficient in isolation. This framing is essential for interpreting biomarkers as indicators of trajectory and endotype, and for designing precision immunomodulation trials that match therapeutic direction, dose, and timing to a patient’s evolving immune course (3, 11, 14, 21).

**Table 2** summarizes the two dominant interpretive schools and presents a reconciliatory, endotype- and time-window-aware synthesis that is used throughout this review (3, 10, 14, 16, 17).

**Table 2:** Debates & reconciliations (storm-centric vs suppression-centric).

Dimension	Storm-centric view	Suppression-centric view	Reconciliatory synthesis (this review)
Core claim	Early uncontrolled inflammation is the primary engine of organ injury and early death	Immunoparalysis/exhaustion drives secondary infection, chronic critical illness, and late mortality (often present early)	Sepsis is trajectory + endotype driven: hyperinflammation and low-response programs can be parallel, with timing/compartment heterogeneity
Main supporting evidence types	Cytokine/alarmin networks; endothelial injury; immunothrombosis; early physiological collapse	mHLA-DR downshift; lymphopenia; checkpoint up-regulation; impaired ex vivo cytokine production; infection/viral reactivation links	Multi-cohort transcriptomic endotypes + single-cell state dictionaries + longitudinal switching patterns
Explanatory power	Explains shock/ARDS/microvascular collapse and why early resuscitation/source control matter	Explains late infections, viral reactivation, prolonged ICU course, PICS-like phenotypes	Explains clinical heterogeneity and trial failures (wrong patient, wrong direction, wrong time)
Key limitations	Tends to assume linear “storm→later suppression”; underestimates early low-response states	Risks treating suppression as uniform/late; may underweight tissue injury risks in storm-dominant endotypes	Requires dynamic monitoring; a single marker/time point can misclassify direction
Therapeutic implication	“Brake” inflammation early (but unstratified trials often neutral)	“Boost” immunity/release inhibitory brakes (but risk in storm-dominant)	Bidirectional, time-stamped, endotype-matched immunomodulation; serial re-typing and composite endpoints

This table contrasts two dominant interpretive schools in sepsis immunopathology—storm-centric and suppression-centric—and summarizes a reconciliation used throughout this review. “Supporting evidence types” indicates the typical study modalities emphasized by each view (e.g., cytokine/endothelial injury readouts vs. immune-function or cellular-state measures), rather than an exhaustive bibliography. The “reconciliatory synthesis” frames sepsis as a time-window- and endotype-dependent immune trajectory in which hyperinflammation and immunosuppression may occur in parallel; thus, therapeutic implications should be interpreted as directionally conditional on patient state and sampling time. PICS, persistent inflammation–immunosuppression–catabolism syndrome; ICU, intensive care unit; mHLA-DR, monocyte human leukocyte antigen-DR.

### 3.4 Unified trajectory/endotype model

“Immune endotypes” provide a unifying hierarchy for the above constructs: biologically anchored patient subgroups defined by shared immune circuitry and mediator patterns. Recent consensus efforts and multi-cohort host-transcriptomic studies have proposed reproducible immune dysregulation frameworks and blood transcriptomic endotypes that capture distinct inflammatory–suppressive balances, trajectories, and outcomes (14, 16, 17). These endotypes are not reducible to severity scores; instead, they represent distinct mechanistic routes through sepsis immunobiology (14, 16, 17). Within this unified model, storm-dominant, suppression-dominant, and mixed endotypes can each present early, persist, or transition over time, offering a mechanistic explanation for clinical heterogeneity and for the repeated failure of unstratified immunomodulation trials (3, 11, 14, 22). Accordingly, therapeutic direction should be endotype- and time-window-aware, aligning immune augmentation or anti-inflammatory strategies with each patient’s evolving trajectory (3, 11, 21, 22).

**Figure 2:**
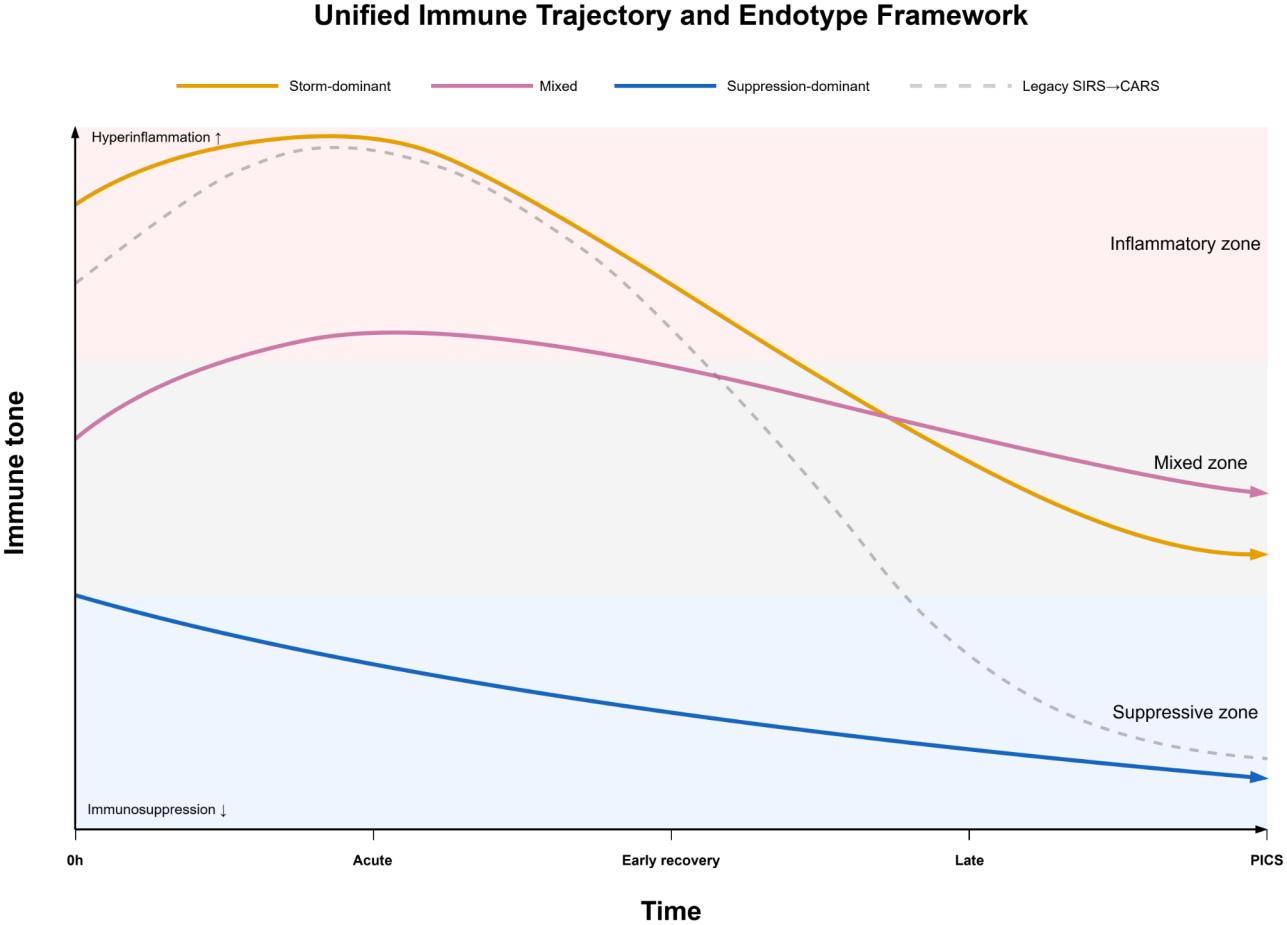
Unified immune trajectory and endotype framework in sepsis. Conceptual schematic illustrating time-resolved immune courses after sepsis onset. The x-axis represents time from presentation (0h) through the acute phase, early recovery, late course, and progression to PICS. The y-axis denotes “immune tone,” ranging from hyperinflammation (upper region) to low response/immunosuppression (lower region). Three prototypical trajectories are shown: a storm-dominant course with early cytokine-driven overactivation followed by partial resolution; a mixed course characterized by concurrent inflammatory and suppressive programs; and a suppression-dominant course with early skewing toward immune paralysis and progressive deepening of low-response states. Shaded horizontal bands indicate corresponding inflammatory, mixed, and suppressive endotype zones. The dashed curve depicts the legacy systemic inflammatory response syndrome to compensatory anti-inflammatory response syndrome (SIRS→CARS) biphasic model for contrast. This unified framework emphasizes early endotype divergence, parallel activation–suppression biology, and the need for trajectory-guided interpretation of biomarkers and precision immunomodulation. SIRS, systemic inflammatory response syndrome; CARS, compensatory anti-inflammatory response syndrome; PICS, persistent inflammation–immunosuppression–catabolism syndrome.

To orient the reader, **Figure 2** summarizes the unified, time-resolved immune-trajectory and endotype framework that underpins the mechanisms and precision-therapy discussions below.

For a time-resolved orientation linking mechanisms to bedside decision windows, a pragmatic 0 h–weeks timeline is provided in **Supplementary Table 1**.

## 4 Early hyperinflammation and immune overactivation

### 4.1 Landscape of early immune overactivation

Early sepsis is characterized by a rapid innate immune surge that is essential for pathogen control but can also initiate immune “reprogramming” that shapes later trajectories in many patients (7, 11, 19). Neutrophils expand within hours through demargination and emergency granulopoiesis, then enter highly activated states marked by degranulation, oxidative burst, and pattern-recognition signaling (7, 19). These early neutrophil waves are heterogeneous: alongside antimicrobial effector programs, single-cell and multi-omic studies identify subsets biased toward amplified inflammation and tissue injury (7, 21). Neutrophil extracellular trap (NET) formation (NETosis) is a prominent feature of this phase; chromatin extrusion and granule protein deposition can trap microbes, but released histones and proteases can injure endothelium and propagate organ damage when clearance is inadequate (19, 21). Neutrophil–platelet–endothelium crosstalk further amplifies these effects, generating immunothrombosis that links innate defense to microvascular obstruction, impaired perfusion, and thromboinflammatory organ dysfunction (3, 7, 19).

Monocytes and macrophages are simultaneously driven into high-output inflammatory programs through PAMP and DAMP sensing, resulting in early cytokine release and inflammasome engagement (3, 19, 21). Inflammasome-dependent interleukin (*IL*)-1 family signaling intensifies local and systemic inflammation and, together with metabolic stress, pushes mononuclear phagocytes toward phenotypes that later display reduced antigen-presenting capacity (19, 21, 23). Complement activation—especially via complement component 5a—adds another layer of early amplification by enhancing neutrophil recruitment, endothelial activation, and cytokine production (3, 19). Yet excessive or sustained complement component 5a signaling can dysregulate leukocyte function and worsen collateral tissue injury, underscoring its double-edged role (19, 21).

**Figure 3:**
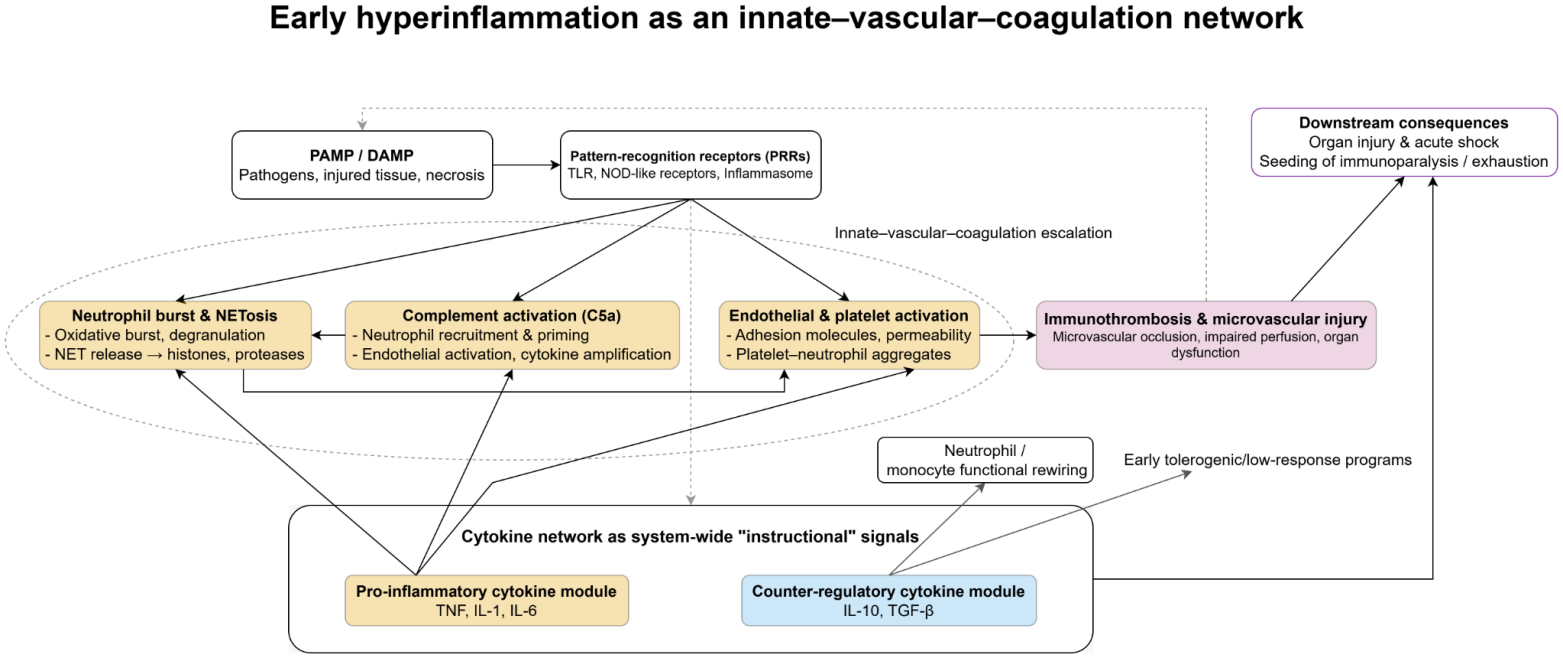
Early hyperinflammation as an innate–vascular–coagulation network. Pathogen- and damage-associated molecular patterns (PAMP/DAMP) activate pattern-recognition receptors (PRRs; Toll-like receptors, NOD-like receptors, inflammasomes), which in turn drive neutrophil burst and NETosis, complement activation (C5a), and endothelial/platelet activation. Crosstalk among these modules amplifies thrombo-inflammatory injury and culminates in immunothrombosis and microvascular dysfunction. In parallel, pro-inflammatory cytokines (*TNF, IL-1, IL-6*) and counter-regulatory cytokines (*IL-10, TGF-β*) form system-wide “instructional” modules that sustain innate effector programs while initiating early tolerogenic/low-response rewiring, thereby coupling acute organ injury with the seeding of later immunoparalysis and immune exhaustion. PAMP, pathogen-associated molecular pattern; DAMP, damage-associated molecular pattern; PRR, pattern-recognition receptor; TLR, Toll-like receptor; NOD, nucleotide-binding oligomerization domain; NET, neutrophil extracellular trap; NETosis, neutrophil extracellular trap formation; C5a, complement component 5a; TNF, tumor necrosis factor; IL, interleukin; TGF-β, transforming growth factor-β.

Taken together, early hyperinflammation is best understood not as a single linear cascade but as an integrated innate–vascular–coagulation network in which immune cells are functionally biased rather than uniformly activated (3, 11, 19). The direction and intensity of that bias—toward microbial resistance versus tissue toxicity—vary across patients and help set the stage for later immune exhaustion, antigen-presentation defects, and chronic critical illness (5, 11, 14, 21). **Figure 3** summarizes this network architecture.

### 4.2 “Instructional” effects of cytokine networks on immune-cell fate

The early inflammatory milieu instructs immune-cell fate through coordinated cytokine networks rather than isolated mediators (3, 4, 11). Systems-level analyses in sepsis suggest that specific pairwise and higher-order cytokine combinations encode system-wide transcriptional and cellular responses, with threshold behavior and feed-forward loops capable of rapidly shifting immune states (4). In practice, tumor necrosis factor/*IL-1*/*IL-6*–dominant patterns can sustain myeloid inflammatory effector programs, while parallel *IL-10*/transforming growth factor beta and stress-associated cytokines drive suppressive rewiring within the same early window (3, 7, 19). This network logic helps explain why hypercytokinemia and immunosuppressive phenotypes often coexist at presentation (7, 11, 19).

A recent critical review of cytokines in sepsis similarly emphasized that cytokine-mediated systemic inflammation is closely linked to multiple organ dysfunction and that cytokine activity should be interpreted as dynamic interacting networks rather than isolated mediators (24).

In adaptive immunity, early cytokine overload intersects with antigen burden and neuroendocrine stress to drive profound lymphocyte apoptosis, particularly among cluster of differentiation 4-positive (*CD4+*) and *CD8+* T cells (7, 19, 23). Surviving lymphocytes frequently progress along exhaustion trajectories characterized by checkpoint upregulation and diminished effector cytokine production, contributing to early loss of pathogen-specific function despite ongoing innate activation (19, 20, 23). These dynamics emerge early enough to support mixed/parallel activation–suppression models rather than a late-phase “switch” (3, 11, 23).

Myeloid fate is likewise instructed by the cytokine environment. Emergency granulopoiesis and accelerated myelopoiesis replenish circulating cells, but they can also skew differentiation toward immature neutrophil and monocyte populations with altered transcriptional programs (7, 21). Multi-omic atlases show that these immature myeloid subsets can be simultaneously hyperinflammatory and immunosuppressive—exhibiting high tissue-injury potential while dampening T-cell activity and antigen presentation (7, 21). In monocytes and dendritic cells, early exposure to combined inflammatory and counter-regulatory cytokines promotes reduced HLA-DR expression, impaired interferon signaling, and metabolic constraints, yielding antigen-presentation defects that are detectable early in the course (3, 7, 11, 19). Thus, early hyperinflammation should be viewed as instructional: it imprints durable cellular states through coupled cytokine “codes” that can seed or accelerate immunoparalysis even as storm signals remain active, depending on host endotype and timing (4, 11, 14, 21).

**Figure 4:**
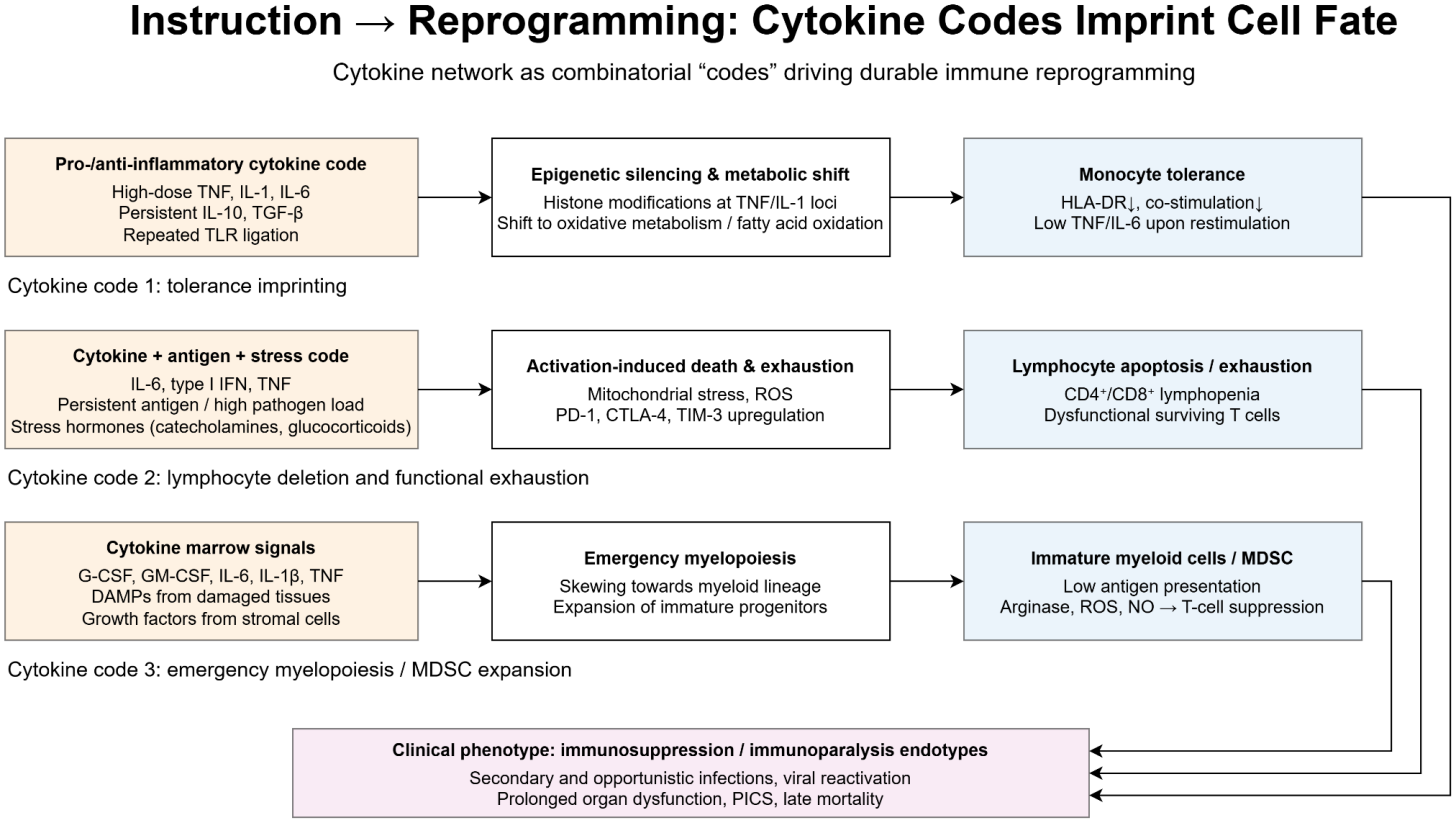
Instruction → reprogramming: cytokine codes imprint cell fate. Cytokines act as combinatorial “instructional codes” that durably reprogram innate and adaptive immunity in sepsis. Pro-/anti-inflammatory cytokine codes with high-dose *TNF*, *IL-1* and *IL-6* combined with persistent *IL-10* and *TGF-β* and repeated *TLR* ligation (cytokine code 1) drive epigenetic silencing and metabolic shifts in monocytes, leading to monocyte tolerance with reduced *HLA-DR* and blunted *TNF*/*IL-6* responses. When inflammatory cytokines are integrated with persistent antigen and neuroendocrine stress (cytokine code 2), lymphocytes undergo activation-induced cell death and exhaustion, resulting in *CD4^+^*/*CD8^+^* lymphopenia and dysfunctional surviving T cells. Bone-marrow–directed cytokine signals including *G-CSF, GM-CSF, IL-6, IL-1β* and *TNF*, together with DAMPs and stromal growth factors (cytokine code 3), trigger emergency myelopoiesis and expansion of immature myeloid cells and MDSC. These three cytokine codes converge on clinical immunosuppression/immunoparalysis endotypes characterized by secondary and opportunistic infections, viral reactivation, prolonged organ dysfunction and late mortality. TLR, Toll-like receptor; HLA-DR, human leukocyte antigen-DR; IFN, interferon; G-CSF, granulocyte colony-stimulating factor; GM-CSF, granulocyte–macrophage colony-stimulating factor; DAMP, damage-associated molecular pattern; MDSC, myeloid-derived suppressor cell; ROS, reactive oxygen species; NO, nitric oxide; PICS, persistent inflammation, immunosuppression and catabolism syndrome.

Together, these findings support the view that cytokines function as combinatorial “instructional codes” that durably imprint cell fate rather than merely mediating transient inflammation. **Figure 4** summarizes three archetypal cytokine codes and their downstream reprogramming trajectories in sepsis.

### 4.3 Debate: necessary defense or futile injury?

A persistent debate is whether early hyperinflammation is primarily protective or pathologic. The defense-centric view emphasizes that rapid innate escalation—neutrophil deployment, complement activation, inflammasome signaling, and high cytokine output—is crucial for containing invasive pathogens, and that insufficient early inflammation risks uncontrolled microbial spread and refractory shock (3, 7, 19). This view aligns with guideline priorities that focus on timely antimicrobials, source control, and organ support rather than routine early immunosuppression (1).

The injury-centric view argues that disproportionate innate activation drives collateral damage through NET-driven thromboinflammation, endothelial breakdown, and excessive cytokine networking, and that these processes can promote later immunoparalysis via apoptosis, exhaustion, and suppressive myelopoiesis (4, 7, 19, 21, 22). In this framing, the “storm” and subsequent vulnerability are mechanistically continuous rather than sequential accidents (11, 14, 21).

This interpretation is consistent with cytokine-focused syntheses showing that mediator networks required for host defense can, when excessive or persistent, amplify systemic inflammation and contribute to multiple organ dysfunction (24).

A reconciliatory synthesis supported by recent endotype and trajectory work is that both positions can be correct, depending on time window and host biology (3, 11, 14). Transcriptomic and cellular endotyping suggests that some patients are storm-dominant, others exhibit mixed early activation–suppression, and some are suppression-skewed from onset, with each pattern predicting distinct risks of organ injury versus secondary infection (14, 16, 22). This heterogeneity helps explain why unstratified anti-inflammatory trials have largely failed and why future immunomodulation must be matched to early trajectory and endotype rather than to a single global model of sepsis (3, 11, 14, 22).

Taken together, these debates converge on a practical point: early inflammatory escalation is not only an acute driver of organ injury but also an instructional program that imprints durable suppressive and exhaustion-like states. As a result, many patients begin to drift toward immunoparalysis while hyperinflammatory signals are still present, and the ensuing clinical vulnerability reflects coupled reprogramming rather than a clean phase transition. The next section therefore dissects the cellular and molecular mechanisms of immunoparalysis, starting with monocyte/macrophage deactivation and antigen-presentation failure.

## 5 Transition to immunoparalysis: cellular and molecular mechanisms

Before detailing mechanisms, we clarify usage across this review: immunoparalysis is treated as an umbrella trajectory of sepsis low responsiveness spanning innate and adaptive compartments. Immune exhaustion represents a dominant adaptive/NK cell component within that trajectory, while PICS denotes the chronic critical illness phenotype that emerges when immunoparalysis coexists with low-grade inflammation and sustained catabolism.

For a cell-by-cell overview of immune reprogramming across major compartments, see **Supplementary Table 2**.

### 5.1 Monocyte/macrophage deactivation and antigen-presentation failure

A hallmark of sepsis-induced immunoparalysis is the rapid reprogramming of monocytes and macrophages from early cytokine-producing sentinels into low-response innate states with blunted antimicrobial and antigen-presenting capacity (7, 11, 13, 21). Clinically, this transition is most consistently tracked by reduced surface HLA-DR on circulating monocytes, accompanied by coordinated downregulation of major histocompatibility complex class II transcriptional modules and reduced costimulatory molecules (e.g., *CD80/CD86*), leading to ineffective priming of adaptive immunity (8, 13, 25). Functional assays align with this phenotype: monocytes display attenuated tumor necrosis factor and *IL-6* production after ex vivo lipopolysaccharide restimulation, a pattern widely described as endotoxin tolerance and interpreted as an actively regulated state of restrained responsiveness rather than passive “cell fatigue” (11, 13, 21, 26). Single-cell profiling further resolves this shift into discrete *HLA-DR*–low, S100A-high suppressive monocyte programs that coexist with inflammatory subsets and predict worse immune competence (8, 13).

The inflammatory environment itself “instructs” monocyte deactivation. Sustained exposure to PAMPs and DAMPs, together with a cytokine balance that progressively favors *IL-10* and transforming growth factor beta, dampens nuclear factor kappa B–dependent effector programs while promoting scavenging, wound-healing, and anti-inflammatory phenotypes (7, 11, 13, 21). In parallel, immunometabolic rewiring—shifts toward oxidative phosphorylation and lipid handling with reduced glycolysis-linked rapid effector output—supports a tolerant transcriptional architecture and constrains antigen processing and presentation (11, 13, 21). These metabolic constraints also reduce the ability of monocytes/macrophages to generate the costimulatory and cytokine “help” required for T helper 1 (Th1) and cytotoxic responses (11, 13, 25).

Epigenetic stabilization adds a memory layer that can lock in these low-response programs. Sepsis induces durable remodeling of histone marks at promoters and enhancers controlling inflammatory cytokines, antigen-presentation genes, and pattern-recognition receptor pathways, and such chromatin changes can persist after clinical resolution of shock (13, 27). Complementary work suggests that altered DNA methylation also contributes to stable monocyte exhaustion/dysregulation after septic challenge, supporting a model in which epigenetic memory maintains tolerance trajectories beyond the initial trigger (13, 28). Recent conceptual syntheses emphasize that this innate memory is best framed as tolerance-skewed reprogramming, distinct from classical trained immunity that enhances future responses; in sepsis, prior inflammatory stress can “train” monocytes toward restraint (13, 26–28). Mechanistically, this durable deactivation compromises pathogen recognition and antigen relay to lymphocytes, thereby increasing susceptibility to secondary bacterial and fungal infections and raising late mortality risk in susceptible trajectories (11, 13, 25).

### 5.2 Lymphocyte depletion and T-cell exhaustion

Adaptive immune failure in sepsis reflects both quantitative lymphocyte loss and qualitative fate reprogramming. Marked lymphopenia develops early through apoptosis and pyroptosis of cluster of differentiation 4–positive and *CD8+* T cells, B cells, and NK cells, driven by high inflammatory cytokine exposure, antigen excess, and neuroendocrine stress (29, 30). The depth of depletion is clinically consequential, but recovery is often incomplete because proliferative renewal is impaired, homeostatic cytokine signaling is disrupted, and thymic output declines during sustained critical illness (7, 13, 30). As a result, sepsis survivors can remain in a prolonged adaptive “valley,” even when innate cell counts rebound (11, 13, 30).

Surviving lymphocytes are biased toward dysfunctional lineages. Integrative reviews of contemporary patient data describe skewing from Th1-type antimicrobial programs toward Th2–dominant and regulatory phenotypes, with disturbed Th17/regulatory T cell balance and relative expansion of forkhead box P3-positive (*FOXP3+*) regulatory T cells in many immune trajectories (7, 13, 21, 30). Such differentiation shifts reduce interferon gamma (*IFN-γ*)–mediated bacterial and viral control, weaken mucosal and barrier defense, and strengthen counter-regulatory loops that reinforce monocyte tolerance (7, 13, 21, 30). Cytotoxic arms are also reprogrammed: *CD8+* T cells and NK cells show impaired degranulation, reduced granzyme/perforin activity, and diminished functional avidity toward infected targets (7, 13, 30). Importantly, these changes reflect regulated fate decisions under inflammatory constraint, not merely loss of cell numbers (11, 30).

A central qualitative layer is T-cell exhaustion. Sepsis induces early and sustained upregulation of inhibitory receptors, most prominently programmed cell death 1 (*PD-1*) on effector T cells, alongside increased programmed death-ligand 1 (*PD-L1*) on monocytes, neutrophils, and endothelial cells (13, 31, 32). Engagement of this axis arrests proliferation, suppresses cytokine transcription, and imposes metabolically constrained low-output states that resemble exhaustion in chronic infections, but in sepsis can arise within days (31–33). Cytotoxic T-lymphocyte–associated protein 4 (*CTLA-4*) enrichment—particularly on regulatory T cells and exhausted effectors—further limits costimulation and *IL-2*–dependent renewal (13). T-cell immunoglobulin and mucin-domain containing-3 (*TIM-3*) signaling has emerged as another sepsis-relevant inhibitory pathway that can suppress Th1 and cytotoxic T-lymphocyte (CTL) programs and consolidate suppressive trajectories (13, 34). Broad reviews suggest that other inhibitory receptors, including T-cell immunoreceptor with Ig and ITIM domains (TIGIT), likely contribute to deeper exhaustion in some patients, although their relative importance appears context- and endotype-dependent (7, 13). Together, inhibitory receptors, transcriptional silencing, and metabolic insufficiency make exhaustion a plausible active contributor to immunoparalysis rather than merely a marker (13, 31–34).

Clinically, depletion plus exhaustion helps explain hallmark late-sepsis vulnerabilities—reactivation of latent viruses, higher rates of nosocomial infection and invasive fungal disease, and increased late mortality—even in patients who no longer exhibit overt hyperinflammatory shock (11, 13, 30). These outcomes support a trajectory model in which early inflammation can imprint a prolonged adaptive low-output state that often persists without timely reversal (11, 13, 21, 30).

### 5.3 Myeloid reprogramming and stress hematopoiesis

Late sepsis often displays a quantity–function paradox in innate immunity: neutrophil and monocyte counts may be preserved or rebound, yet antimicrobial effectiveness deteriorates (7, 11, 13, 21). Stress hematopoiesis is a major driver. Cytokine-rich environments promote emergency granulopoiesis and myelopoiesis, releasing immature neutrophils and monocytes into circulation with transcriptional programs distinct from mature effectors (7, 13, 21). Recent single-cell atlases and trajectory syntheses suggest that these immature cells often combine high inflammatory potential with impaired chemotaxis and reduced microbicidal precision, making them simultaneously injurious and ineffective (7, 13). In the Nature Immunology endotype framework, emergency granulopoiesis is not merely compensatory; it can propel an extreme response trajectory enriched for suppressive myeloid states (7).

Concurrently, sepsis expands suppressive myeloid phenotypes, including myeloid-derived suppressor cells and low-density neutrophils (7, 13, 21). These populations inhibit T-cell proliferation, deplete arginine and other nutrients required for lymphocyte function, and secrete *IL-10*–family mediators that reinforce adaptive exhaustion and monocyte deactivation (7, 13, 21). Single-cell atlases also place MDSC-like programs along broader myeloid continua, suggesting that suppressive phenotypes emerge through progressive reprogramming rather than a fixed lineage (7, 13).

Functionally, these reprogrammed states undermine host defense. Immature or suppressive neutrophils show reduced directional migration and impaired phagocytosis, with dysregulated reactive oxygen species production and NET release that can perpetuate organ injury without improving clearance (7, 13, 21). Monocytes in these trajectories maintain low *HLA-DR* and defective antigen presentation, weakening the innate–adaptive relay and sustaining endotoxin tolerance (8, 13, 25). The net effect is an innate compartment that is numerically abundant but qualitatively biased away from efficient pathogen control, helping explain secondary infections despite neutrophilia and linking stress hematopoiesis to chronic critical illness (5, 7, 11, 13).

### 5.4 Immune checkpoints and negative regulatory axes

Checkpoint signaling provides a convergent negative-regulatory layer that translates inflammatory stress into durable immunoparalysis. The *PD-1*/*PD-L1* pathway is central: *PD-1* rises on exhausted cluster of differentiation 4–positive and *CD8+* T cells, while *PD-L1* increases on monocytes, neutrophils, and endothelial cells, creating inhibitory synapses that suppress proliferation, reduce *IFN-γ* and tumor necrosis factor output, and impair cytotoxicity (13, 31–33). In innate cells, *PD-1* or *PD-L1* engagement can also blunt phagocytosis and antigen presentation, linking checkpoint biology directly to monocyte low-response states (13, 31, 32). These effects position *PD-1*/*PD-L1* as a functional driver of both adaptive and innate paralysis, not simply a correlate of severity (31–33).

T cell immunoglobulin and mucin-domain containing 3 has emerged as an additional driver within this inhibitory landscape. Sepsis-focused studies describe T cell immunoglobulin and mucin-domain containing 3 upregulation on T cells and myeloid cells, with signaling that dampens Th1 and CTL programs, limits *IFN-γ* output, and promotes macrophage tolerance (13, 34). Together with *CTLA-4*–enriched regulatory circuits and other inhibitory receptors highlighted in broader sepsis immunology reviews, these checkpoints form layered inhibitory axes that actively reshape cell states rather than merely reporting disease intensity (7, 13, 34). Conceptually, checkpoints convert an acute inflammatory “alarm” into durable cellular restraint, closing the loop between early storm signaling and later refractory hyporesponsiveness (13, 31–34).

These biology-first insights help explain why checkpoint blockade has re-entered therapeutic debate, while also underscoring why stratification is essential. Releasing inhibitory circuits may benefit suppression-dominant trajectories, but in storm-dominant endotypes it could amplify tissue injury or precipitate immune-mediated harm (11, 13, 21, 31, 32). Thus, checkpoint pathways sit at the interface of mechanistic understanding and precision immunomodulation trial design (11, 13, 31–34).

### 5.5 Key challenge: defining immunoparalysis as a dynamic syndrome

Immunoparalysis cannot be captured by a single laboratory snapshot. Low-response programs evolve over days to weeks, differ between blood and tissue compartments, and map to distinct immune endotype trajectories (11, 13). Single time-point biomarkers may therefore miss parallel activation–suppression states or misclassify patients whose immune course is shifting (11, 13, 21). A dynamic framing that integrates timing, compartment, and trajectory is essential before biomarkers can guide bedside decisions and precision trials, motivating the endotyping and immune-monitoring sections that follow (11, 13).

Section 5 shows that immunoparalysis is not an endpoint but a trajectory; Section 6 extends this discussion by focusing on chronic critical illness, high-dimensional endotyping, and the translational debates they have triggered.

## 6 Persistent inflammation–immunosuppression and immune endotypes

### 6.1 PICS concept and clinical phenotype

PICS describes a post-sepsis trajectory of chronic critical illness in which the host fails to return to immune homeostasis and instead stabilizes in an altered “late-sepsis” state (5, 35). Conceptually, PICS is defined by a triad: (i) persistent low-grade systemic inflammation, (ii) sustained immune suppression with impaired host defense, and (iii) catabolic and metabolic frailty reflected by ongoing protein breakdown, sarcopenia, and failure to regain physiologic reserve (5, 35, 36). Clinically, this triad presents as prolonged ICU dependence, recurrent or opportunistic infections, poor wound healing, muscle wasting, neurocognitive decline, and ultimately reduced long-term quality of life among survivors (37, 38).

**Figure 5:**
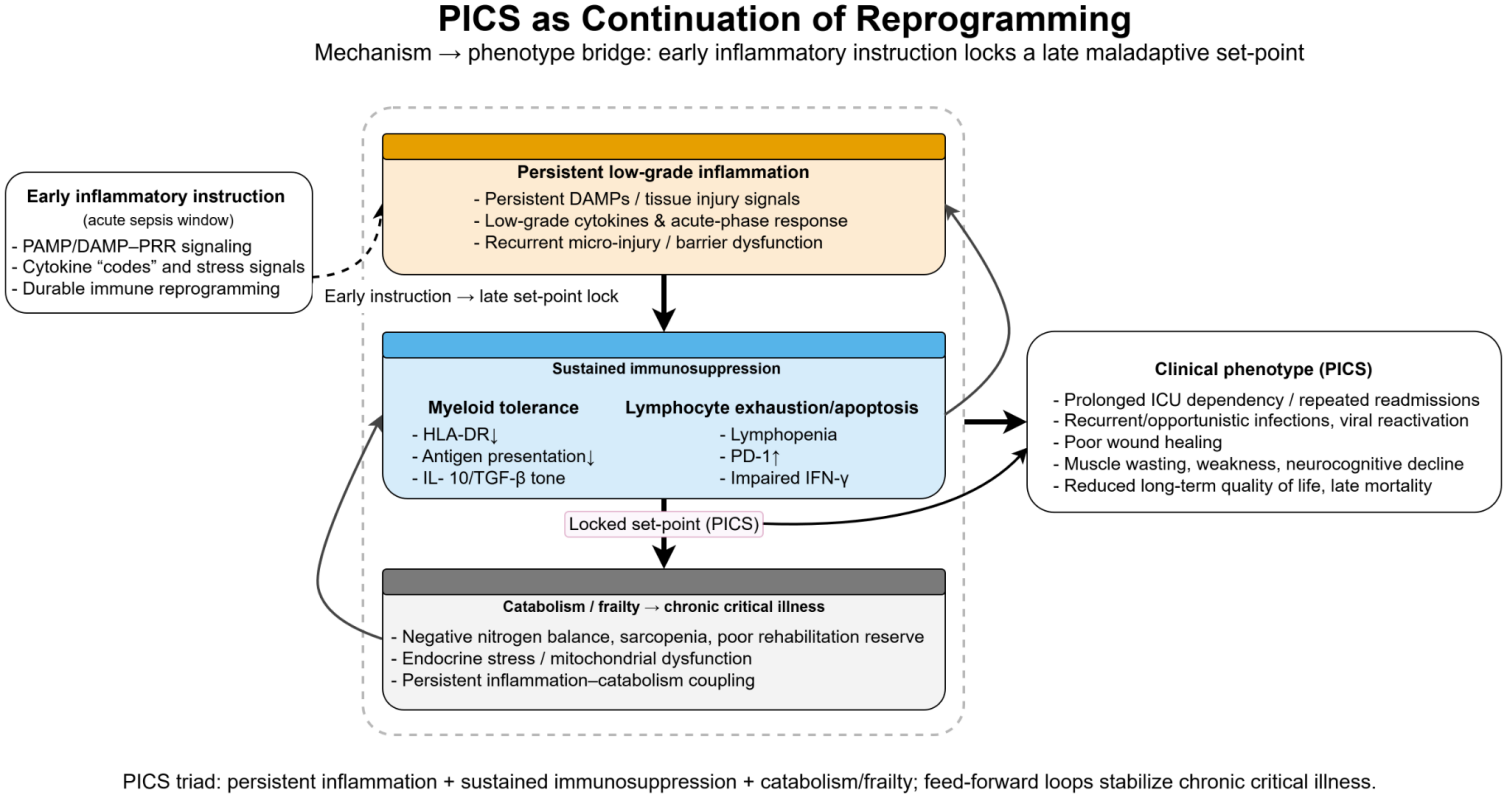
PICS as continuation of immune reprogramming (mechanism–phenotype bridge). Persistent inflammation–immunosuppression and catabolism syndrome (PICS) is depicted as a stabilized late maladaptive set-point emerging from early inflammatory instruction. The triad is shown as layered domains—persistent low-grade inflammation, sustained immunosuppression (myeloid tolerance and lymphocyte exhaustion/apoptosis), and catabolism/frailty leading to chronic critical illness—linked by directional arrows. Feed-forward loops illustrate how secondary infections/viral reactivation can reinforce inflammation and how catabolic frailty can limit immune recovery, thereby sustaining the PICS state. The right panel summarizes downstream clinical consequences, including prolonged ICU dependency, recurrent infections, impaired healing, muscle wasting, neurocognitive decline, and late mortality. DAMP, damage-associated molecular pattern; HLA-DR, human leukocyte antigen-DR; ICU, intensive care unit; IFN-γ, interferon-gamma; IL, interleukin; PD-1, programmed cell death protein 1; PICS, persistent inflammation–immunosuppression and catabolism syndrome; PRR, pattern-recognition receptor; TGF-β, transforming growth factor-beta.

To bridge definition and mechanism, **Figure 5** summarizes how early inflammatory “instruction” can lock in a maladaptive late set point that links the PICS triad to bedside phenotypes.

PICS is increasingly anchored to measurable immune states rather than treated solely as a descriptive label. A 2025 single-cell study identified post-sepsis immune-cell signatures consistent with PICS that combine persistent myeloid inflammatory programs with *HLA-DR*–low suppressive monocytes and exhaustion-like features in adaptive compartments, implying that PICS represents continuity of immune reprogramming rather than a distinct recovery phase (38). This view reframes late sepsis: even when hemodynamics normalize and infection appears controlled, the immune system may remain locked in a maladaptive set point that helps explain recurrent infections and slow functional recovery (5, 35, 38).

Notably, current Surviving Sepsis Campaign guidance standardizes acute resuscitation and antimicrobial care but offers limited direction on endotype-informed immune monitoring or long-term immunorecovery strategies—leaving a clinical gap precisely where PICS operates (1). PICS therefore provides a practical lens for interpreting chronic critical illness as an immune–metabolic syndrome rooted in earlier inflammatory instruction (5, 35, 38).

### 6.2 Single-cell/spatial multi-omics and immune endotypes

High-dimensional profiling over the past five years has made immune heterogeneity the central empirical fact in sepsis biology. Single-cell RNA sequencing and cellular indexing of transcriptomes and epitopes by sequencing resolve circulating leukocytes into discrete activation and suppression states that can coexist early, diverge across organs, and persist into chronic trajectories such as PICS (9, 13, 38). For example, single-cell RNA sequencing atlases across bacterial sepsis subtypes repeatedly identify parallel expansion of inflammatory myeloid programs alongside tolerant *HLA-DR*–low monocytes, while lymphocyte compartments are often depleted or biased toward exhaustion-like states (13, 38). Together, these patterns undermine one-directional “storm-then-paralysis” models and support mixed, time-dependent state landscapes (13, 38). Consistent with this, a 2025 review integrating single-cell RNA sequencing, single-cell assay for transposase-accessible chromatin sequencing, and spatial datasets argues that “immune low-response states” are not a single entity; instead, they lie along myeloid and lymphoid continua in which distinct regulatory circuits dominate across patients and time windows (13).

**Figure 6:**
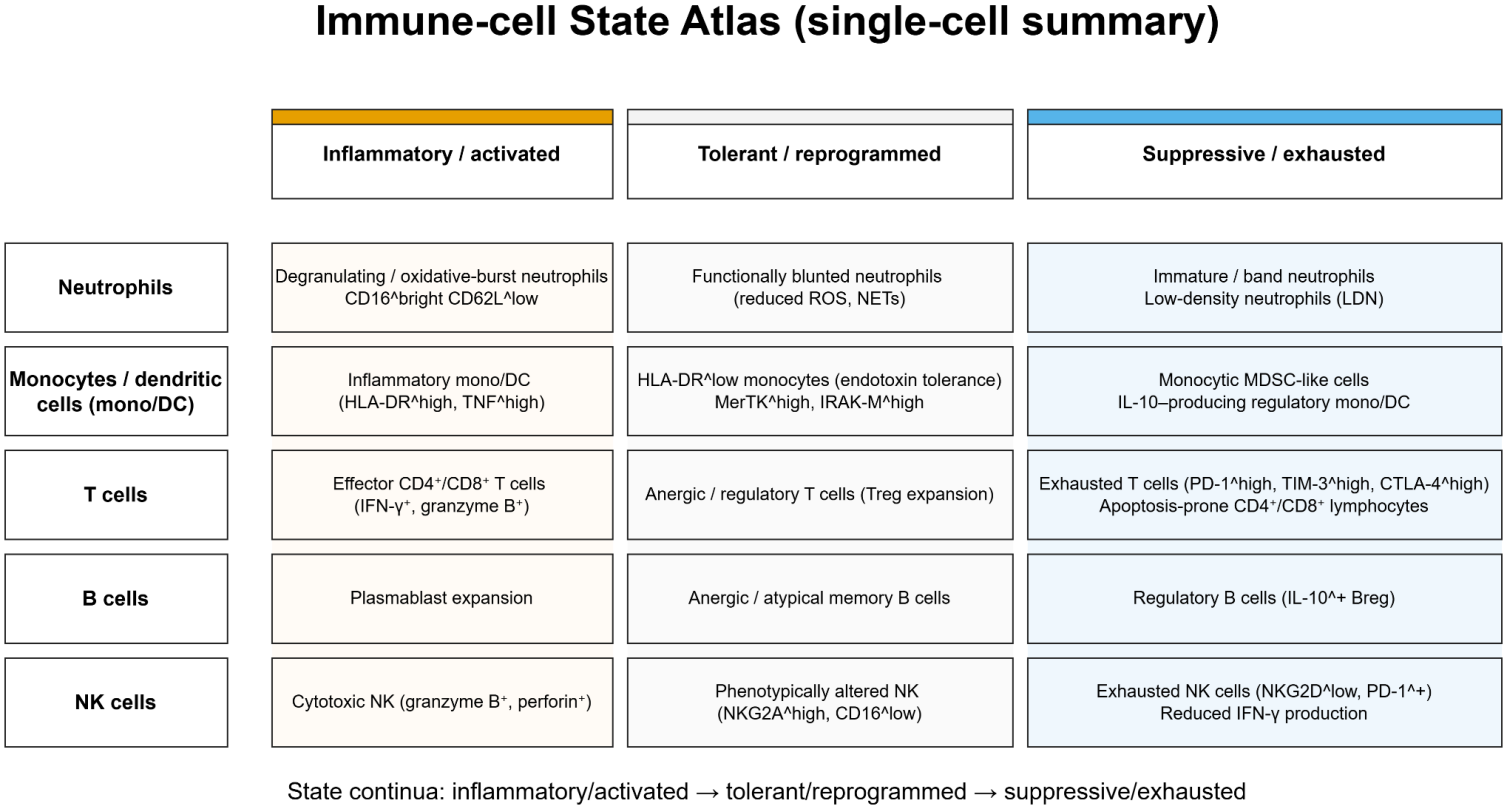
Immune-cell state atlas (single-cell summary). Schematic atlas summarizing recurrent immune-cell states reported by single-cell transcriptomics and high-dimensional cytometry in sepsis. Rows represent major leukocyte lineages (neutrophils, monocytes/dendritic cells, T cells, B cells, and NK cells), and columns depict a functional continuum from inflammatory/activated through tolerant/reprogrammed to suppressive/exhausted states. Canonical sepsis-associated examples include degranulating/oxidative-burst neutrophils, functionally blunted neutrophils and immature/low-density neutrophils; inflammatory monocytes/DC versus *HLA-DR* low “tolerant” monocytes and MDSC-like suppressive myeloid states; effector T cells versus regulatory/anergic states and exhausted/apoptosis-prone T cells; plasmablast expansion alongside dysfunctional memory and regulatory B-cell states; and NK-cell phenotypes ranging from cytotoxic activation to altered and exhausted states. This atlas illustrates that immune endotypes in sepsis can be grounded in a measurable dictionary of lineage-specific cell states rather than undifferentiated “immune dysregulation”. DC, dendritic cell; mono/DC, monocytes/dendritic cells; ROS, reactive oxygen species; NETs, neutrophil extracellular traps; LDN, low-density neutrophils; HLA-DR, human leukocyte antigen-DR; TNF, tumor necrosis factor; IFN-γ, interferon-gamma; PD-1, programmed cell death protein 1; TIM-3, T-cell immunoglobulin and mucin-domain containing-3; CTLA-4, cytotoxic T-lymphocyte–associated protein 4; Treg, regulatory T cell; MDSC, myeloid-derived suppressor cell; Breg, regulatory B cell; MerTK, MER tyrosine kinase; IRAK-M, interleukin-1 receptor–associated kinase M; NK, natural killer; NKG2A/NKG2D, natural killer group 2A/2D; IL, interleukin.

Collectively, these findings support a measurable “cell-state dictionary” that underpins immune endotypes in sepsis; **Figure 6** presents a simplified atlas of canonical lineage-specific states across inflammatory → tolerant → suppressive/exhausted continua.

Beyond cataloging cell states, high-dimensional profiling has clarified how stress hematopoiesis and myeloid skewing actively shape trajectories. The Nature Immunology endotype study showed that emergency granulopoiesis generates immature neutrophil programs that are simultaneously hyperinflammatory and suppressive, linking specific myeloid state compositions to extreme-risk trajectories (7). This suggests that late immunoparalysis can be seeded early through marrow “instruction,” not only through peripheral exhaustion (7).

Bulk and multi-cohort transcriptomics now converge with single-cell findings to define portable immune endotypes with outcome relevance. A Communications Medicine study spanning global cohorts identified reproducible host gene-expression endotypes, showing that these groupings capture cross-population heterogeneity better than traditional clinical labels (39). A 2025 multi-cohort RNA sequencing meta-analysis further distilled sepsis into discrete endotypes with dominant coagulopathic, inflammatory, or adaptive-immune features, each associated with distinct risks and therapeutic implications (22). Complementing this, a Cell Reports Medicine study framed sepsis outcome as a balance between transcriptional programs of antimicrobial resistance and systemic inflammation, emphasizing that prognosis depends on the relative strength and timing of these programs rather than any single pathway (40).

Spatial transcriptomics and emerging three-dimensional tissue approaches add organ context and reinforce that immune states are compartment-specific (13). For example, lung and kidney injury niches may exhibit intensified tolerance-associated myeloid signatures and barrier-linked inflammatory crosstalk that peripheral blood sampling does not fully capture (13). Longitudinal multi-omics enables trajectory models in which patients move through inflammatory, tolerant, exhausted, or reparative state spaces over time (7, 13, 22, 38, 40). These models help explain why mixed programs can persist into PICS and why late outcomes cannot be inferred from early severity alone (38, 40).

To make endotypes clinically tangible, **Supplementary Table 3** synthesizes recurring immune endotype patterns reported across multi-cohort transcriptomics and high-dimensional cellular atlases, highlighting core modules, dynamics, outcome links, and simplified bedside proxies.

### 6.3 Debate: which axis of typing is most clinically useful?

With endotypes established, a central controversy is which dimension should anchor clinically actionable typing. One school favors cell-composition phenotyping (e.g., proportions of immature neutrophils, *HLA-DR*–low monocytes, exhausted T cells), arguing that single-cell state dictionaries map directly onto mechanisms and drug targets (7, 13). Another emphasizes portable bulk gene-expression signatures, because multi-cohort transcriptomics can stratify patients robustly across regions, pathogens, and analytic platforms (22, 39, 40). A third approach prioritizes functional immune tests—such as ex vivo cytokine production or antigen-presentation capacity—because these provide bedside-proximal readouts of immune competence (11, 13, 25).

In practice, mismatches across systems are common: a patient may map to a transcriptomic endotype suggestive of suppression while still exhibiting mixed cellular states in blood, or may shift substantially over days (13, 22, 39). Cell-state systems offer mechanistic clarity but are harder to deploy at scale in urgent care pathways; bulk signatures are portable but may blur organ-specific biology; functional assays are clinically intuitive but often capture only selected arms of immunity (11, 13, 22). The emerging balanced view is that no single axis is sufficient—hybrid, time-stamped approaches that integrate cellular composition, transcriptional programs, and functional readouts are most likely to enable precision sepsis care (11, 13, 39).

### 6.4 Current gaps and methodological challenges

Despite rapid progress, multi-omic endotyping faces major translational barriers. Serial sampling is still limited, tissue access is scarce, and many signatures lack prospective, multicenter validation under real-world treatment confounding (11, 13, 22). Therapies, comorbidities, and pathogen diversity can shift immune states, complicating causal inference and cross-study harmonization (13, 40). Peripheral biomarkers remain imperfect proxies for organ immune microenvironments, and scalable pipelines that convert endotypes into trial eligibility rules and on-therapy decision thresholds are not yet mature (11, 13). Addressing these gaps is essential to translate heterogeneity from a descriptive fact into a therapeutic tool.

## 7 Consequences and immune monitoring biomarkers

### 7.1 Secondary and opportunistic infections

The transition to immunoparalysis has direct clinical consequences because reprogrammed innate and adaptive cells lose coordinated pathogen control while remaining metabolically and transcriptionally constrained. In practice, ICU–acquired bacterial and fungal infections cluster among patients who follow suppression-dominant or mixed immune trajectories: monocyte deactivation limits antigen presentation and Th1 instruction, while lymphocyte exhaustion weakens cytotoxic and helper functions, slowing clearance of new pathogens and allowing colonization to progress to invasive disease (11, 21). This creates a feedback loop: secondary infection is not only an outcome of immunoparalysis but also a potent amplifier of it, because each infectious “hit” adds PAMP and DAMP load that reinforces tolerant myeloid programs and checkpoint-biased lymphocyte states. Late sepsis is therefore best understood as iterative immune stress layered on an already reprogrammed baseline, rather than a clean recovery phase (11, 21).

Opportunistic infections and latent virus reactivation further illustrate this mechanism–outcome coupling. Reactivation of herpesviruses—such as cytomegalovirus or herpes simplex virus—is increasingly recognized in critically ill patients with sepsis, particularly when lung injury or prolonged ventilation coexists, consistent with impaired T cell and NK cell surveillance (41–43). However, causality remains debated: viral reactivation may directly worsen organ injury and infection risk, but it may also function as a severity-linked biomarker of deeper immunoparalysis rather than an independent driver (41, 42). Either way, the signal is clinically informative—reactivation tends to occur on immunosuppressive trajectories and can flag patients in whom adaptive exhaustion and myeloid tolerance are dominant (11, 41).

### 7.2 Organ injury and immune–organ crosstalk

Immune reprogramming reshapes the pattern and persistence of organ injury because immune, endothelial, coagulation, and metabolic networks are tightly coupled in sepsis. Early hyperinflammation initiates thromboinflammatory and barrier-disruptive circuits, but as immune cells shift toward tolerant or exhausted states, they do not simply “turn off.” Instead, they adopt biased programs that can sustain microvascular dysfunction, impair repair, and promote maladaptive remodeling (44). This implies that organ failure is not a passive by-product of inflammation; it is a systems-level output of dysregulated immune–organ crosstalk in which immune tone, perfusion, and tissue metabolism coevolve over time (44).

The lung provides a clear example. In septic acute respiratory distress syndrome, neutrophil–endothelial interactions and immunothrombosis drive early alveolar damage, while later myeloid tolerance and impaired antigen presentation can limit pathogen clearance and promote secondary pneumonia. Thus, the same reprogramming that dampens systemic immune performance can prolong local injury and heighten infection risk within the lung niche (11, 44). Similarly, kidney injury reflects compartment-specific immune states: cytokine-primed monocytes and neutrophils infiltrate peritubular microenvironments and may transition into low-response or suppressive phenotypes that impair efferocytosis and resolution, sustaining endothelial leak and metabolic stress (44). Across organs, tissue niches also reciprocally sculpt immune phenotypes—hypoxia, coagulation products, and cell-death metabolites reinforce tolerant transcriptional programs—so immune remodeling and organ dysfunction can become mutually stabilizing features of late sepsis (11, 44).

### 7.3 Immune monitoring biomarkers and risk stratification

Risk stratification in late sepsis sits at the tension between real-world operability and mechanistic prediction. Bedside-feasible markers such as monocyte human leukocyte antigen-DR (*mHLA-DR*) and absolute lymphocyte count are attractive because they are scalable and inexpensive (25, 30, 45). They also map onto core reprogramming axes—innate tolerance and adaptive depletion/exhaustion—making them conceptually aligned with trajectory biology (25, 30, 45). Ferritin, as a widely available acute-phase reactant that tracks macrophage- and cytokine-linked inflammatory load, can serve as a pragmatic proxy for storm-dominant endotypes and thus complements low-response markers in composite stratification (45, 46). Functional readouts (e.g., ex vivo cytokine production capacity) add biological meaning by testing whether cells remain responsive rather than merely present (45, 46). Yet these accessible measures are imperfect when used in isolation because sepsis trajectories are mixed, time-dependent, and compartment-specific (11, 45).

Time-stamped monitoring is therefore essential. Longitudinal analyses show that immunoparalysis biomarkers vary across days, and the same numeric value can reflect different biology in early versus late windows (15, 25, 47). For example, a low value may represent transient suppression during shock recovery or persistent tolerant programming that predicts secondary infection (11, 47). This is why a single cutoff at one time point cannot reliably define immune state. Static thresholds also ignore trajectory switching, organ-specific divergence, and endotype context. Serial trends and combinations—*mHLA-DR* with absolute lymphocyte count, plus a functional assay when feasible—are more likely to discriminate recovery of immune competence from consolidation of immunoparalysis (46, 47).

**Table 3** provides a practical summary of candidate biomarkers for dynamic immune monitoring, including biological interpretation, recommended sampling windows, trend-based interpretation, and bedside feasibility (15, 25, 45–47).

**Table 3:** Biomarkers for dynamic immune monitoring (what to measure, when, and how to read trends).

Biomarker/readout	What it tracks (biology)	Suggested sampling	Trend interpretation (prefer trends over cutoffs)	Bedside feasibility
mHLA-DR (flow)	Monocyte antigen presentation; innate competence/immunoparalysis	Admission/0–24h; Day 3; Day 5–7; re-check with new deterioration	Persistently low/falling → sustained immunoparalysis & infection risk; rising → immune recovery	Moderate (flow + standardization)
ALC (CBC-diff)	Adaptive “mass”; lymphocyte attrition	Baseline; daily D1–7; then 2–3×/week if prolonged ICU	Non-resolving lymphopenia → vulnerability (secondary infection/viral reactivation); rebound supports recovery (may lag function)	High (routine)
Ferritin	Macrophage activation/inflammatory load (context-sensitive)	Baseline; q48–72h in acute phase; repeat if flare suspected	Rapid rise/very high → hyperinflammatory signal; downtrend → storm resolving	High (common), but low specificity
CRP ± PCT (pragmatic control markers)	Inflammatory/infectious burden (not immune competence)	Daily early; then spaced	Failure to fall → ongoing driver (infection/inflammation); falling does *not* exclude immunoparalysis → pair with ALC/mHLA-DR	High
PD-1 (T)/PD-L1 (mono) (flow)	Checkpoint inhibition; exhaustion-/suppression-leaning environment	Often most informative Day 3–7; repeat in prolonged/recurrent infection	Up-trending with persistent lymphopenia/low mHLA-DR → exhaustion-like risk; down-trending may align with recovery	Moderate (flow; gating expertise)
Functional assay: ex vivo LPS→TNF (whole blood)	Myeloid functional reserve; tolerant low-response state	Baseline if feasible; Day 3–5; repeat if suspected immunoparalysis	Blunted & persistent → functional immunoparalysis, recovering response → regaining competence	Low–Moderate (specialized)
Endotype signature (transcriptomic/module-based)	Mechanistic subgrouping; integrates storm vs suppression programs	Day 0–1 ± Day 3 (serial “re-typing” in platforms)	Endotype + shift over time can guide “brake vs boost” framing and trial enrichment	Low (research-grade/turnaround limits)
Clinical consequence signal: secondary infection/viral reactivation (PCR where used)	Real-world readout of failed host defense (validates trajectory)	Trigger-based; weekly in prolonged/high-risk ICU (local protocols)	New infections/reactivation despite source control → clinically meaningful immune dysfunction	Moderate (protocol-dependent)

Use serial composites (e.g., mHLA-DR + ALC ± ferritin, plus infection/organ-failure slopes) rather than any single marker at a single time point; interpret trends in the context of steroids, transfusions, liver injury, and marrow-suppressive therapies. CRP/PCT are control markers rather than direct immune-competence readouts. Representative supporting evidence is cited in the corresponding text sections. mHLA-DR, monocytic HLA-DR expression; ALC, absolute lymphocyte count; CBC-diff, complete blood count with differential; CRP, C-reactive protein; PCT, procalcitonin; PD-1, programmed cell death protein 1; PD-L1, programmed death-ligand 1; LPS, lipopolysaccharide; TNF, tumor necrosis factor; PCR, polymerase chain reaction; ICU, intensive care unit.

**Figure 7:**
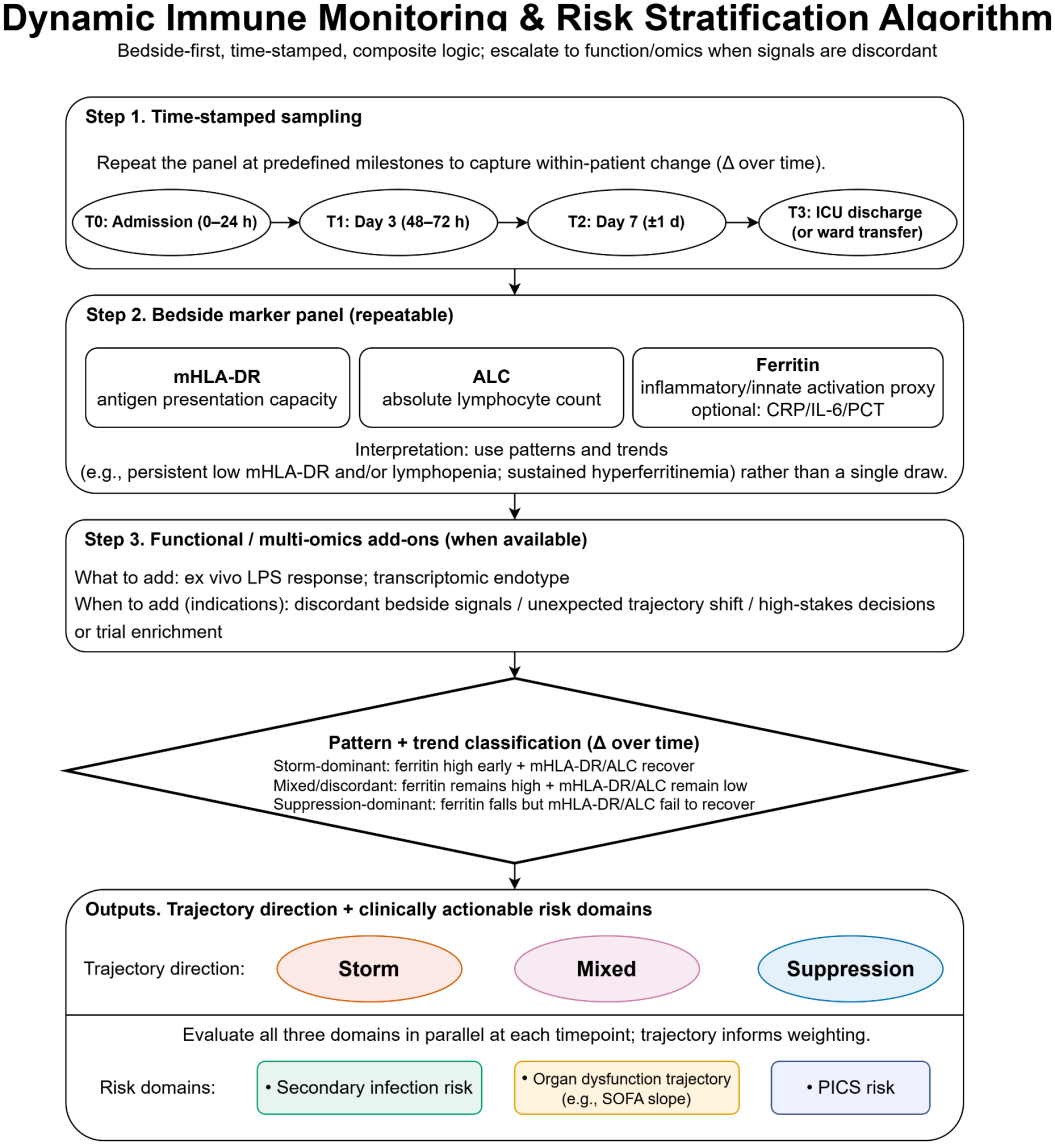
Dynamic immune monitoring and risk stratification algorithm (bedside-first, time-stamped, composite interpretation). This schematic outlines a pragmatic, repeatable workflow for sepsis immune re-phenotyping across predefined milestones (admission, Day 3, Day 7, and ICU discharge/ward transfer). A compact bedside panel (monocytic *HLA-DR*, absolute lymphocyte count, and ferritin, with optional infection/inflammation control markers) is interpreted as patterns and trends rather than single time-point cutoffs. Functional testing and transcriptomic endotyping are added when bedside signals are discordant, trajectories shift unexpectedly, or higher-stakes decisions require deeper resolution. Trajectory classification (storm-dominant, mixed/discordant, or suppression-dominant) then informs weighting of three clinically actionable risk domains—secondary infection risk, organ dysfunction trajectory, and persistent inflammation–immunosuppression–catabolism syndrome risk—evaluated in parallel at each timepoint. ICU, intensive care unit; mHLA-DR, monocytic human leukocyte antigen–DR expression; ALC, absolute lymphocyte count; CRP, C-reactive protein; IL-6, interleukin-6; PCT, procalcitonin; LPS, lipopolysaccharide; SOFA, Sequential Organ Failure Assessment; PICS, persistent inflammation–immunosuppression–catabolism syndrome.

To operationalize this dynamic, composite approach at the bedside, **Figure 7** presents a time-stamped immune-monitoring and risk-stratification algorithm that integrates repeatable markers with optional functional and multi-omic add-ons.].

Omics-derived signatures add a higher-resolution layer by identifying endotype-linked transcriptomic or multiplex immune patterns that can forecast trajectories beyond traditional laboratory tests (11, 14, 15). The emerging consensus is that monitoring should be dynamic and composite: integrating bedside markers with trajectory-aware endotype clues will likely outperform any single biomarker (11, 46). Future directions include rapid bedside immune-function platforms, real-time updating of endotype classification as trajectories evolve, and trial designs that enroll or adapt therapy based on evolving immune state rather than static labels (45–47).

## 8 Therapeutic implications and debates

### 8.1 Why “anti-inflammation alone” largely failed

For decades, sepsis trials attempted to blunt inflammation in a uniform manner, yet most yielded neutral or negative results. The reasons map directly onto the modern immune-reprogramming framework. First, sepsis is not a single inflammatory state; it spans mixed and rapidly shifting immune trajectories (3, 11, 48). Without endotype stratification, the same anti-inflammatory intervention may benefit storm-dominant patients but harm suppression-dominant patients, and these opposing effects can cancel out in unselected cohorts (3, 49, 50). Second, timing is decisive: hyperinflammation and immunosuppression often emerge in parallel and can pivot quickly (11, 21, 48). If an intervention is delivered even slightly late, it may suppress a response that is already drifting toward tolerance or exhaustion, thereby deepening immunoparalysis and increasing secondary infection risk (11, 48, 49).

Third, many anti-inflammatory strategies targeted single mediators or narrow pathways. Yet cytokine and danger-signal networks in sepsis are redundant and nonlinear, and they can reroute around a blocked node (4, 48). As a result, inhibiting one target rarely resets the system-level immune program across innate, adaptive, and stromal compartments (11, 48, 49). Finally, many trials implicitly assumed that the “storm” was the dominant driver of organ injury. This framing overlooked early low-response myeloid states, lymphocyte attrition, and checkpoint-biased exhaustion that are already emerging in many patients at presentation (7, 13, 21). In other words, treating inflammation alone often meant treating the wrong biology, in the wrong patient, at the wrong time.

These failures are therefore informative: they imply that sepsis therapy must be bidirectional and trajectory-aware, using immune phenotypes and time windows to decide whether to dampen runaway inflammation or to restore immune competence (11, 49, 50).

### 8.2 Immune boosting vs immune braking: choosing direction

A precision view reframes immunotherapy as a choice of direction based on endotype and time-stamped monitoring, rather than a menu of drugs. When bedside and functional signals indicate an immunoparalysis-leaning trajectory—such as falling *mHLA-DR*, progressive lymphopenia, or weak *IFN-γ*–type effector signatures—immune-boosting strategies become biologically coherent (12, 49, 51). In this setting, agents such as granulocyte–macrophage colony-stimulating factor, *IFN-γ*, or *IL-7* are best conceptualized as state “resetters”: granulocyte–macrophage colony-stimulating factor may restore antigen presentation and cytokine responsiveness in *HLA-DR*–low myeloid programs, while *IL-7* and *IFN-γ* aim to improve adaptive survival, proliferation, and antimicrobial effector tone (12, 49, 51). Trials and scoping syntheses report consistent immunologic “wake-up” signals and acceptable short-term safety, but clinical outcome benefits remain uncertain and require larger, endotype-guided randomized controlled trials (18, 49, 50).

Conversely, releasing inhibitory brakes (checkpoint-based disinhibition) targets negative regulatory axes that actively maintain low-output states. The *PD-1*/*PD-L1* pathway is upregulated across T-cell and myeloid compartments in sepsis and can suppress proliferation, cytokine production, and phagocytic competence, making it a plausible driver rather than a passive marker (31–33). Checkpoint modulation therefore offers a mechanistic route to recover effector function and potentially reduce secondary infections in suppression-dominant endotypes (31, 32, 49). However, this same approach may be risky in storm-dominant or *IFN-γ*–high trajectories, where removing inhibitory constraints could intensify tissue injury or immunothrombosis (11, 21, 49).

In short, the direction of immunomodulation should be chosen using endotype plus serial monitoring. A one-size-fits-all boost—or a blanket release of inhibitory brakes—is mechanistically inconsistent with parallel trajectories and is likely to recapitulate past trial failures (3, 49, 50).

To translate this bidirectional principle into actionable options, **Table 4** maps representative therapeutic candidates to immune trajectory/endotype and time window, with key evidence signals and safety cautions (1, 12, 18, 45, 49).

**Table 4:** Therapeutic candidates mapped to endotype + time window (boost vs brake).

Intervention/strategy	Direction (boost/brake)	Target/mechanism	Best-fit trajectory + time window (who/when)	Evidence (brief)	Key risks/watch-outs
Source control + antimicrobials (+ de-escalation)	Foundation	Removes pathogen drive (upstream of storm/suppression)	All trajectories; ASAP (0–24h); reassess Day 3–5	GC/RCT-informed	Under/over-treatment, MDR, microbiome injury; improvement ≠ immune recovery
Hemodynamic optimization (fluids/vasopressors; avoid overload)	Indirect brake	Limits endothelial/organ injury while maintaining perfusion	Storm-dominant or mixed; 0–24h, then dynamic	GC/RCT-informed	Fluid overload vs under-resuscitation; needs frequent re-titration
Corticosteroids (shock context)	Mixed (brake > may worsen suppression)	Broad anti-inflammatory + vasoactive support	Vasopressor-refractory shock, early shock window (not “endotype therapy”)	RCT/GC	Hyperglycemia, delirium, secondary infection risk; confounds leukocyte-based markers
Targeted cytokine blockade (anti–IL-1/anti–IL-6/anti-TNF, etc.)	Brake	Dampens specific inflammatory nodes	Strict storm-enriched subsets, *very early*; generally experimental in sepsis	Mostly negative/mixed RCT history; subset hypotheses	Timing/heterogeneity mismatch; infection risk; harms if suppression already present
Extracorporeal mediator modulation (hemoadsorption, etc.)	Brake (non-specific)	Removes circulating mediators (± endotoxin)	Proposed for high-burden storm, early window; center-protocol dependent	Mixed/controversial	Non-selective removal (incl. drugs); uncertain target engagement; resource intensive
IL-7	Boost	Lymphocyte survival/expansion; restores adaptive “mass”	Suppression-/lymphopenia-predominant, typically Day 3–7+	Phase-2/translational	Inflammatory flare, capillary leak concerns; needs phenotype + monitoring
Myeloid stimulants (GM-CSF ± IFN-γ)	Boost	Improves antigen presentation/macrophage killing; raises competence signals	HLA-DR-low + refractory infections, usually Day 3–7+	Small RCTs/case-series signals	Can amplify inflammation if too early; leukocytosis/hemodynamic instability; careful timing
Checkpoint modulation (anti–PD-1/anti–PD-L1)	Boost	Releases inhibitory synapses; restores T/NK function	Exhaustion-/checkpoint-high, often Day 3–7 or later, esp. recurrent/secondary infections	Early-phase/experimental	Immune over-activation/autoimmunity; dangerous if storm biology still dominant

This table is intended as a conceptual mapping of therapeutic direction, immune trajectory, and time window rather than a treatment recommendation. “Boost” refers to immune-restorative approaches for suppression-dominant or low-response states, whereas “brake” refers to anti-inflammatory or mediator-limiting approaches for storm-dominant states. Candidate interventions should be interpreted in the context of serial immune monitoring, infection control, organ support, and patient-specific contraindications. ASAP, as soon as possible; GC, guideline consensus (or guideline-concordant care); RCT, randomized controlled trial; MDR, multidrug resistance; ARDS, acute respiratory distress syndrome; AKI, acute kidney injury; IL, interleukin; IL-7, interleukin-7; GM-CSF, granulocyte–macrophage colony-stimulating factor; IFN-γ, interferon-gamma; PD-1, programmed cell death protein 1; PD-L1, programmed death-ligand 1; ICU, intensive care unit.

**Figure 8:**
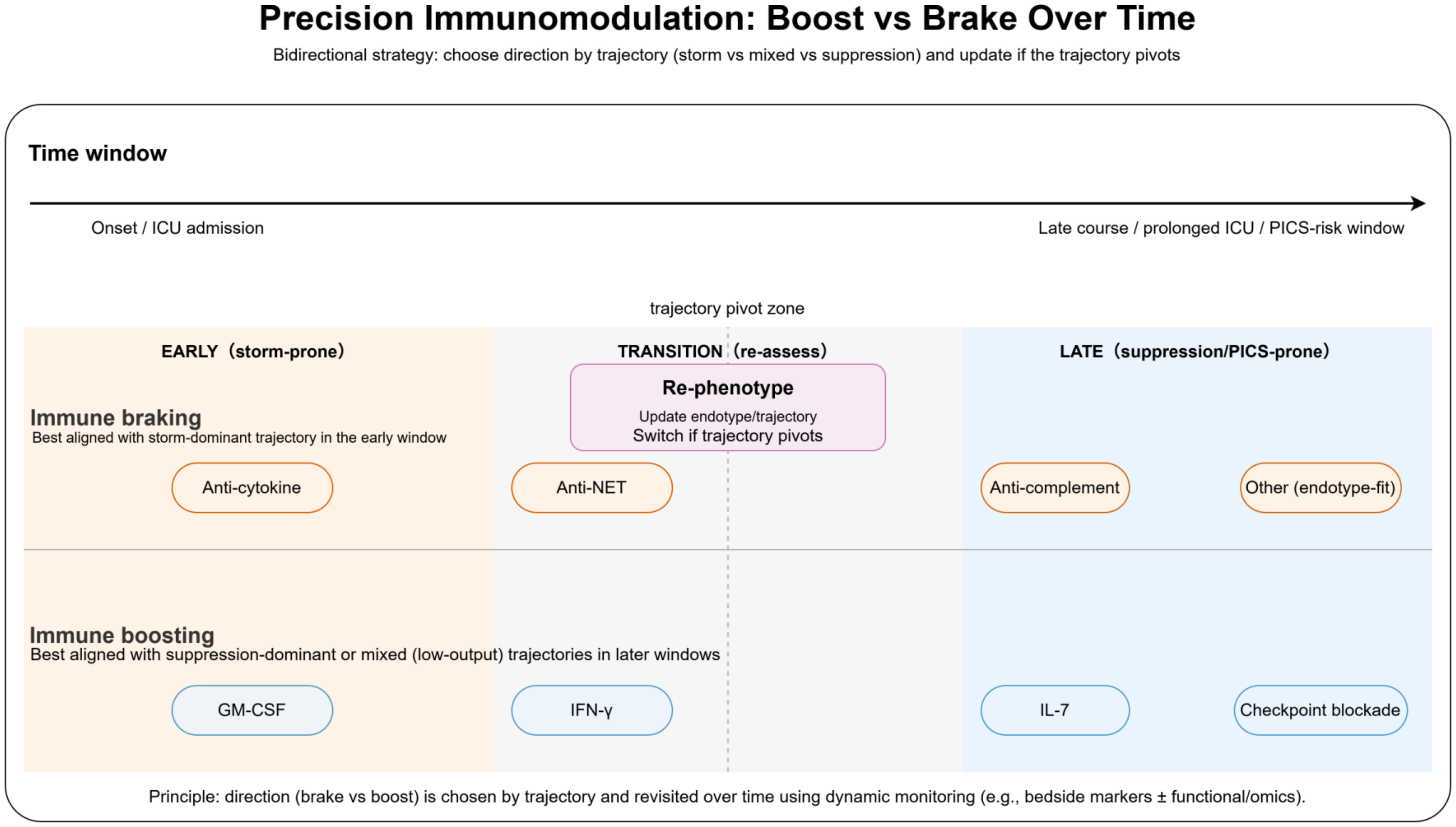
Precision immunomodulation: boost vs brake over time. Conceptual schematic of bidirectional, trajectory-guided immunomodulation across the sepsis course. The upper panel illustrates immune braking strategies (e.g., anti-cytokine, anti-NET, anti-complement, and other endotype-fit anti-inflammatory/anti-immunothrombotic approaches) that are most coherent in an early, storm-prone window. The lower panel illustrates immune boosting strategies (e.g., *GM-CSF, IFN-γ, IL-7*, and checkpoint blockade) that are more aligned with later suppression-dominant or mixed low-output states, including PICS-risk windows. A central re-phenotype/switch checkpoint emphasizes iterative reassessment using dynamic monitoring (serial bedside markers ± functional/omics profiling) and switching direction when the immune trajectory pivots, addressing timing/endotype mismatch that likely contributed to failures of one-direction-for-all trials. ICU, intensive care unit; PICS, persistent inflammation, immunosuppression, and catabolism syndrome; NET, neutrophil extracellular trap; GM-CSF, granulocyte–macrophage colony-stimulating factor; IFN-γ, interferon-gamma; IL-7, interleukin-7.

**Figure 8** translates this principle into a time-aware, bidirectional schematic: early storm-prone patients may require immune “braking,” whereas later suppression-leaning or mixed low-output states may benefit from immune “boosting,” with rephenotyping and switching if the trajectory pivots.

### 8.3 How future trials should be designed

Next-generation sepsis immunotherapy trials should treat immune reprogramming as the design backbone rather than a *post hoc* explanatory layer. Enrollment should be guided by immune endotypes or biomarker composites—rather than clinical severity alone—such as ferritin–*mHLA-DR* patterns, transcriptomic endotypes, or functional stimulation tests that distinguish low-response from hyperinflammatory states (45, 46, 50). Because trajectories evolve, trials should incorporate serial reassessment and adaptive logic, using platform or response-adaptive designs to switch, escalate, or stop immunomodulation as immune states change (46, 50). Endpoints should also be trajectory-aligned: secondary infection burden, organ-function slopes, and longer-term recovery or quality-of-life outcomes more directly capture the consequences of immune reprogramming than 28-day mortality alone (45, 50). Finally, multicenter validation and linkage to real-world monitoring pipelines are essential to ensure that endotypes remain portable across pathogens, health systems, and treatment contexts (11, 50).

## 9 Discussion & conclusion

### 9.1 Integrating schools of thought: a balanced synthesis

Across this review, the core message is straightforward: sepsis is not a single-phase disorder. It is a time- and endotype-driven disease of immune trajectories, in which early inflammatory “instruction” reshapes subsequent host defense and recovery.

The storm-centric school explains how uncontrolled innate activation, endothelial injury, and thromboinflammation precipitate acute organ failure and early death. Its main limitation is an implicit linearity that underestimates immunosuppressive programs that can emerge during the “storm.”

The immunosuppression-centric school shows how monocyte deactivation, lymphocyte loss, and checkpoint-driven exhaustion contribute to secondary infections, chronic critical illness, and late mortality. Its limitation is treating suppression as a uniform late phase rather than a variable, context-modulated trajectory.

We therefore adopt a reconciliatory framework: distinct endotypes traverse different—and sometimes concurrent—hyperinflammatory and low-response states within narrow time windows. This unified view explains divergent bedside phenotypes, requires dynamic interpretation of biomarkers, and clarifies why prior “one-direction, one-time-window” trials failed to deliver consistent benefit (7, 14, 49).

### 9.2 Key unresolved problems

Three unresolved problems now define the translational bottleneck. First, dynamic immune monitoring remains difficult because immune states can shift within hours, are strongly confounded by therapies (e.g., antibiotics, steroids, ventilation, transfusion), and differ across organ compartments; consequently, a single measurement can misclassify trajectory direction and lead to inappropriate immunomodulation. Second, endotype stability and reproducibility remain uncertain: transcriptomic or cellular clusters may be robust at the cohort level yet unstable within individuals over time or across platforms, limiting portability and clinical confidence in endotype-based decisions. Third, the evidence base for individualized timing and direction of therapy is still thin; without prospective proof that “boosting” or “braking” matched to trajectory improves outcomes, precision immunotherapy risks repeating the failures of unstratified anti-inflammatory trials (11, 46, 50).

### 9.3 Current research gaps

Key research gaps are not merely descriptive—they directly obstruct clinical translation. Large, multicenter, longitudinal cohorts that sample patients across acute sepsis, ICU recovery, and post-sepsis syndromes remain scarce, leaving trajectory models underpowered and biased toward early inpatient windows. Mapping between peripheral biomarkers and tissue- or organ-level immune networks is also incomplete: blood signatures only partially reflect lung, kidney, marrow, or brain microenvironments where immune reprogramming and injury co-evolve, complicating inference of organ-specific biology from feasible bedside tests. Finally, real-world evidence for treatment guided by immune typing remains limited, with few pragmatic trials that integrate serial immune measures into actionable pathways. Together, these gaps delay implementation of endotypes, degrade trial precision in enrollment and timing, and ultimately constrain improvements in secondary infection prevention and long-term functional recovery (11, 13, 46, 50).

### 9.4 Future directions

Several feasible directions follow logically from the immune-reprogramming model. First, single-cell and multi-omics discoveries should be distilled into clinically actionable endotypes that are transferable across settings, mechanistically interpretable, and reproducible over time; achieving this will require standardized feature sets and cross-platform calibration so endotypes can be updated serially rather than assigned once (13, 22, 39). Second, bedside immune-function platforms and dynamic composite scores—combining myeloid activation/tolerance, lymphocyte competence, and functional responsiveness—should replace reliance on single static thresholds and enable time-window–specific interpretation (11, 46, 47).

Third, precision immunomodulation must span the full clinical course—from acute shock through PICS-like recovery—so that “boosting” or “braking” can be adjusted as trajectories evolve rather than fixed at admission. Fourth, adaptive platform trials with immune-endotype–guided enrollment, mandated rephenotyping, and trajectory-aligned composite endpoints (secondary infection burden, organ-function slopes, and long-term quality of life) offer the most direct path toward “type first, treat second” sepsis care (49, 50).

### 9.5 Conclusion

Sepsis outcomes reflect inflammation-driven immune reprogramming that shapes secondary infection risk and long-term recovery. The next stage of progress requires endotype-based, time-stamped monitoring linked to precision immunomodulation—not one-size-fits-all trials. In practical terms, this means translating multi-omics insights into a small, repeatable bedside monitoring set that captures myeloid activation/tolerance, lymphocyte competence, and functional responsiveness, interpreted within prespecified time windows rather than as static cutoffs. These monitoring frameworks should be validated in large, multicenter longitudinal cohorts that span acute sepsis through ICU recovery and post-sepsis syndromes to ensure transferability and temporal stability. Finally, the resulting readouts must be operationalized in endotype-guided adaptive platform trials with mandated rephenotyping and trajectory-aligned composite endpoints—secondary infection burden, organ-function slopes, and long-term quality of life—so success is defined by fewer late infections and meaningful functional recovery, not short-term survival alone.
