# Supplementary material for: Inflammation-driven immune reprogramming in sepsis: from cytokine storm to immunoparalysis
Source: Front Immunol. 2026 Jul 9;17:1887033. doi: 10.3389/fimmu.2026.1887033 (PMC13391346; doi:10.3389/fimmu.2026.1887033)
Supplement: Supplementary file 1 [file SupplementaryFile1.docx]

**Supplementary Methods. Electronic search strategies and record accounting**

**S1. Databases, dates, and limits**

Databases searched:

- MEDLINE via PubMed
- Embase via Embase.com
- Scopus

Search structure: Two complementary electronic searches were run in each database:

- **Search A (mechanistic/phenotyping core):** sepsis + immune reprogramming/trajectory/endotype + single-cell/transcriptomics/multi-omics terms.
- **Search B (translation/monitoring/intervention core):** sepsis + immunoparalysis/immune suppression/exhaustion + immune monitoring/biomarkers + immunomodulation/immunotherapy/trial design terms.

Date window: **1 January 2020 to 31 December 2025**.
Limits: **Humans and English where available** (database filter availability varies by platform).

**S2. MEDLINE (PubMed) search strategies and yields**

**PubMed Search A (mechanistic/phenotyping)**

(

"Sepsis"[Mesh] OR "Septic Shock"[Mesh] OR sepsis[tiab] OR "septic shock"[tiab]

)

AND

(

"immune reprogram*"[tiab] OR reprogram*[tiab] OR

immunoparalysis[tiab] OR "immune paralysis"[tiab] OR

immunosuppress*[tiab] OR "immune suppression"[tiab] OR

"immune exhaustion"[tiab] OR endotype*[tiab] OR phenotype*[tiab] OR

trajector*[tiab] OR "immune trajector*"[tiab] OR

"PICS"[tiab] OR "persistent inflammation immunosuppression catabolism"[tiab]

)

AND

(

"single cell"[tiab] OR scRNAseq[tiab] OR "single-cell"[tiab] OR

transcriptom*[tiab] OR "gene expression"[tiab] OR multiomic*[tiab] OR "multi-omics"[tiab] OR

"mass cytometry"[tiab] OR CyTOF[tiab] OR "spatial transcriptom*"[tiab]

)

AND (english[la])

AND (humans[mh])

AND ("2020/01/01"[dp] : "2025/12/31"[dp])

**PubMed Search B (translation/monitoring/intervention)**

(

"Sepsis"[Mesh] OR "Septic Shock"[Mesh] OR sepsis[tiab] OR "septic shock"[tiab]

)

AND

(

immunoparalysis[tiab] OR immunosuppress*[tiab] OR "immune suppression"[tiab] OR

"immune exhaustion"[tiab] OR "HLA-DR"[tiab] OR "monocyte HLA-DR"[tiab] OR

"secondary infection*"[tiab] OR "viral reactivation"[tiab]

)

AND

(

biomarker*[tiab] OR "immune monitoring"[tiab] OR immunophenotyp*[tiab] OR endotyp*[tiab] OR

"precision immunotherap*"[tiab] OR immunomodulat*[tiab] OR

"GM-CSF"[tiab] OR "granulocyte macrophage colony-stimulating factor"[tiab] OR

"interferon gamma"[tiab] OR IFNg[tiab] OR "IL-7"[tiab] OR

"PD-1"[tiab] OR "PD-L1"[tiab] OR checkpoint*[tiab] OR

"trial design"[tiab] OR "enriched enrollment"[tiab] OR "adaptive trial"[tiab]

)

AND (english[la])

AND (humans[mh])

AND ("2020/01/01"[dp] : "2025/12/31"[dp])

**PubMed yields:** Search A = **272**; Search B = **405**; within-database de-duplication (A+B) = **573** unique records.

**S3. Embase (Embase.com) search strategies and yields**

**Embase Search A (mechanistic/phenotyping)**

(

'sepsis'/exp OR 'septic shock'/exp OR sepsis:ti,ab,kw OR 'septic shock':ti,ab,kw

)

AND

(

'immune reprogramming':ti,ab,kw OR reprogram*:ti,ab,kw OR

immunoparalysis:ti,ab,kw OR 'immune paralysis':ti,ab,kw OR

immunosuppress*:ti,ab,kw OR 'immune suppression':ti,ab,kw OR

'immune exhaustion':ti,ab,kw OR endotype*:ti,ab,kw OR trajector*:ti,ab,kw OR

pics:ti,ab,kw OR 'persistent inflammation immunosuppression catabolism':ti,ab,kw

)

AND

(

'single cell sequencing'/exp OR 'single cell':ti,ab,kw OR 'single-cell':ti,ab,kw OR scrnaseq:ti,ab,kw OR

transcriptom*:ti,ab,kw OR 'gene expression':ti,ab,kw OR multiomic*:ti,ab,kw OR 'multi-omics':ti,ab,kw OR

cytometry:ti,ab,kw OR 'mass cytometry':ti,ab,kw OR cytof:ti,ab,kw OR

'spatial transcriptomics':ti,ab,kw

)

**Embase Search B (translation/monitoring/intervention)**

(

'sepsis'/exp OR 'septic shock'/exp OR sepsis:ti,ab,kw OR 'septic shock':ti,ab,kw

)

AND

(

immunoparalysis:ti,ab,kw OR immunosuppress*:ti,ab,kw OR 'immune suppression':ti,ab,kw OR

'immune exhaustion':ti,ab,kw OR 'human leukocyte antigen dr':ti,ab,kw OR hla-dr:ti,ab,kw OR

'secondary infection':ti,ab,kw OR 'viral reactivation':ti,ab,kw

)

AND

(

biomarker*:ti,ab,kw OR 'immune monitoring':ti,ab,kw OR immunophenotyp*:ti,ab,kw OR endotyp*:ti,ab,kw OR

immunomodulat*:ti,ab,kw OR 'precision immunotherapy':ti,ab,kw OR

'granulocyte macrophage colony stimulating factor':ti,ab,kw OR gm-csf:ti,ab,kw OR

'interferon gamma':ti,ab,kw OR ifn*:ti,ab,kw OR il-7:ti,ab,kw OR

'programmed cell death 1':ti,ab,kw OR pd-1:ti,ab,kw OR pd-l1:ti,ab,kw OR checkpoint*:ti,ab,kw OR

'trial design':ti,ab,kw OR 'adaptive trial':ti,ab,kw OR 'enriched enrollment':ti,ab,kw

)

**Embase yields:** Search A = **541**; Search B = **2,531**; within-database de-duplication (A+B) = **2,620** unique records.

**S4. Scopus search strategies and yields**

**Scopus Search A (mechanistic/phenotyping)**

TITLE-ABS-KEY

(

sepsis OR "septic shock"

)

AND TITLE-ABS-KEY

(

"immune reprogram*" OR reprogram* OR immunoparalysis OR "immune paralysis" OR

immunosuppress* OR "immune suppression" OR "immune exhaustion" OR

endotype* OR trajector* OR PICS OR "persistent inflammation immunosuppression catabolism"

)

AND TITLE-ABS-KEY

(

"single cell" OR "single-cell" OR scRNAseq OR transcriptom* OR "gene expression" OR

multiomic* OR "multi-omics" OR "mass cytometry" OR CyTOF OR "spatial transcriptom*"

)

AND (LIMIT-TO (LANGUAGE, "English"))

AND (PUBYEAR > 2019 AND PUBYEAR < 2026)

**Scopus Search B (translation/monitoring/intervention)**

TITLE-ABS-KEY( sepsis OR "septic shock" )

AND

TITLE-ABS-KEY(

immunoparalysis OR immunosuppress* OR "immune suppression" OR "immune exhaustion"

OR "HLA-DR" OR "monocyte HLA-DR"

OR "secondary infection" OR "secondary infections"

OR "viral reactivation"

)

AND

TITLE-ABS-KEY(

biomarker* OR "immune monitoring" OR immunophenotyp* OR endotyp*

OR immunomodulat* OR "precision immunotherapy"

OR "GM-CSF" OR "interferon gamma" OR IFNg OR "IL-7"

OR "PD-1" OR "PD-L1" OR checkpoint*

OR "trial design" OR "adaptive trial" OR "enriched enrollment"

)

AND (LIMIT-TO (LANGUAGE, "English"))

AND (PUBYEAR > 2019 AND PUBYEAR < 2026)

**Scopus yields:** Search A = **710**; Search B = **1,250**; within-database de-duplication (A+B) = **1,516** unique records.

**S5. Cross-database merge and de-duplication**
Search results were exported in RIS/compatible formats and imported into **EndNote 21** for reference management. De-duplication was performed using EndNote’s duplicate detection workflow (e.g., title/author/year/journal matching) with manual verification where needed. Duplicates were removed first within each database (Search A + Search B) and then across databases after merging all records. After cross-database de-duplication, the combined library contained **3,051** unique records.
