# Supplementary material for: Inflammation-driven immune reprogramming in sepsis: from cytokine storm to immunoparalysis
Source: Front Immunol. 2026 Jul 9;17:1887033. doi: 10.3389/fimmu.2026.1887033 (PMC13391346; doi:10.3389/fimmu.2026.1887033)
Supplement: Supplementary file 2 [file Table1.docx]

**Supplementary Table S1. Timeline of Immune Events (0 h–weeks)**

| **Time window** | **Dominant cells / processes** | **Representative signals (examples)** | **Clinical relevance** |
| --- | --- | --- | --- |
| **0–6 h (recognition–resuscitation window)** | Innate sensing escalation; rapid neutrophil deployment; complement/inflammasome activation; early endothelial–coagulation coupling | High cytokine output and “alarm” programs; early thrombo-inflammatory / barrier-disruptive circuits | Shock/early organ dysfunction risk; therapeutic timing is critical—biology can shift within hours, so interpretation is time-sensitive |
| **6–24 h (acute organ-injury formation)** | Storm-driven tissue injury vs concurrent counter-regulation; onset of “instructional” cytokine coding that imprints later fates | NET-driven thromboinflammation; endothelial breakdown; redundant cytokine networking (injury-centric framing) | Early death/ARDS/AKI risk beginnings; sets up later vulnerability (storm → apoptosis/exhaustion/suppressive myelopoiesis link) |
| **Day 1–3 (early mixed/parallel states become evident)** | Adaptive attrition begins (lymphocyte apoptosis); myeloid reprogramming emerges; immature myeloid subsets appear | Lymphopenia + early exhaustion trajectory signals; reduced antigen-presentation capacity (e.g., early mHLA-DR decline) can be detectable | Supports “mixed/parallel activation–suppression” rather than a late switch; single time-point classification becomes unreliable |
| **Day 3–7 (consolidation vs recovery pivot)** | Checkpoint-driven inhibitory axes consolidate; stress hematopoiesis sustains immature/suppressive myeloid states; organ–immune crosstalk prolongs injury | PD-1/PD-L1 (± TIM-3/CTLA-4) inhibitory synapses; MDSC/LDN expansion; “quantity–function paradox” in innate cells | Rising risk of secondary infections despite “rebounding counts”; divergence across organs (e.g., ARDS: early neutrophil/endothelium injury → later tolerance/poor clearance) |
| **Week 2–4 (late sepsis / prolonged ICU course in some)** | Durable immunoparalysis traits persist; biased repair/remodeling; recurrent insults reinforce tolerant programming | Functional hyporesponsiveness; persistent low HLA-DR / adaptive quiet; latent virus reactivation signals and nosocomial patterns | Late mortality and chronic critical illness risk; highlights need for longitudinal monitoring (trends/slopes) rather than single cutoffs |
| **Weeks to months (PICS-like trajectory; “failure to return to homeostasis”)** | Persistent inflammation + sustained immunosuppression + catabolism/metabolic frailty (PICS triad) | Persistent myeloid inflammatory programs + HLA-DR–low suppressive monocytes + exhaustion-like adaptive features (single-cell anchored view) | Recurrent/opportunistic infections, poor wound healing, muscle wasting, neurocognitive decline, reduced long-term QoL; clinical gap in long-term immunorecovery guidance |

**note:**

Time windows are approximate and intended for orientation rather than rigid staging. Immune activation and low responsiveness frequently overlap, and dominant processes vary by host endotype, infection source, and ongoing care (e.g., antimicrobials, resuscitation, corticosteroids). “Representative signals” are illustrative (not exhaustive) and should be interpreted as trajectories (serial trends) rather than single time-point thresholds.

**Abbreviations:** AKI, acute kidney injury; ARDS, acute respiratory distress syndrome; CTLA-4, cytotoxic T-lymphocyte–associated protein 4; HLA-DR, human leukocyte antigen-DR; ICU, intensive care unit; LDN, low-density neutrophils; MDSC, myeloid-derived suppressor cells; mHLA-DR, monocyte human leukocyte antigen-DR; NET, neutrophil extracellular trap; PD-1, programmed cell death 1; PD-L1, programmed death-ligand 1; PICS, persistent inflammation, immunosuppression, and catabolism syndrome; QoL, quality of life; TIM-3, T-cell immunoglobulin and mucin-domain containing-3.
