# Supplementary material for: Inflammation-driven immune reprogramming in sepsis: from cytokine storm to immunoparalysis
Source: Front Immunol. 2026 Jul 9;17:1887033. doi: 10.3389/fimmu.2026.1887033 (PMC13391346; doi:10.3389/fimmu.2026.1887033)
Supplement: Supplementary file 3 [file Table2.docx]

**Supplementary Table S2. Cell-specific Reprogramming Features**

| **Cell type** | **Abundance shift** | **Functional reprogramming** | **Metabolic / epigenetic features** | **Key pathways** | **Clinical consequences** |
| --- | --- | --- | --- | --- | --- |
| **Monocytes / Macrophages** | Circulating counts may normalize, but **low-responsiveness increases** (e.g., HLA-DR-low subsets). | Reduced antigen presentation; **endotoxin tolerance** (blunted TNF/IL-6 responses to ex vivo stimulation). | Metabolic “energy-saving” setting (reduced rapid-effector programs; relative shift toward oxidative/fatty-acid programs); epigenetic remodeling that stabilizes tolerant memory. | IL-10/TGF-β skew; downregulated MHC-II modules; PD-L1 upregulation contributes to suppressive synapses. | Higher risk of secondary bacterial/fungal infections; persistent immune dysfunction contributing to chronic critical illness/PICS. |
| **Neutrophils** | Neutrophilia is common; **stress hematopoiesis** increases **immature neutrophils / LDNs**. | “Quantity–function paradox”: impaired trafficking precision/phagocytosis despite inflammatory tissue injury; dysregulated ROS/NET balance. | Immaturity-associated transcriptional rewiring; (epigenetic features often inferred and should be detailed in-text if emphasized). | Stress myelopoiesis axis; MDSC-like suppressive continuum; suppression reinforced by PD-1/PD-L1–rich microenvironments. | Contributes to ongoing organ injury while pathogen clearance remains ineffective; aligns with mixed/extreme-risk trajectories. |
| **Dendritic cells (DCs)** | Often decreased in circulation (numerical DC deficiency). | Weakened priming and co-stimulation; reduced interferon-oriented antiviral initiation (tolerogenic bias). | Functional energy limitation and cellular stress programs (conceptual overview; specify platform-specific evidence in-text if needed). | IL-10–dominant tolerogenic circuits; reduced MHC-II/co-stimulatory signaling. | Failed T-cell activation → higher susceptibility to secondary infection and latent viral reactivation (synergizes with T/NK dysfunction). |
| **CD4 T cells** | Early loss via apoptosis/attrition; incomplete recovery in some trajectories. | Reduced Th1 effector support; shift toward Th2/Treg-like programs; impaired helper function. | Metabolic restriction supports low-output states; exhaustion-like biology may emerge with sustained stress. | PD-1 upregulation; additional inhibitory axes (e.g., CTLA-4, TIM-3) can accumulate. | Reduced pathogen control and impaired coordination of adaptive responses; elevated risk of opportunistic infection. |
| **CD8 T cells** | Marked early reduction; recovery may be delayed or incomplete. | Reduced cytotoxic function (degranulation/perforin–granzyme programs); impaired proliferative capacity. | Exhaustion-like low-output setting tied to metabolic insufficiency. | PD-1/PD-L1 axis; TIM-3/CTLA-4 and related checkpoint networks. | Viral reactivation and nosocomial infection risk; contributes to late mortality and prolonged ICU courses. |
| **B cells** | Reduced numbers (apoptosis/attrition). | Diminished antibody responses and antigen-presentation support (expand with data if you emphasize humoral endpoints). | Energy-limited state (high-level summary). | Stress + suppressive cytokine/checkpoint environment shapes dysfunction. | Weaker defense against new pathogens; amplifies risk when combined with T-cell defects. |
| **NK cells** | Reduced counts; impaired surveillance. | Reduced cytotoxicity and IFN-γ output; defective immune monitoring. | Exhaustion-like low-output program (high-level summary). | Checkpoint-rich suppressive networks (often converging on PD-1/PD-L1 signaling context). | Higher risk of latent viral reactivation and secondary infections. |

**Note:**

Entries are intended as a high-level synthesis rather than an exhaustive catalog. “Abundance” and “function” may diverge (e.g., numerical recovery with persistent hyporesponsiveness). Pathways and metabolic/epigenetic features are context- and platform-dependent and should be interpreted alongside timing, infection source, and concurrent therapies.

**Abbreviations:** HLA-DR, human leukocyte antigen–DR; LPS, lipopolysaccharide; LDN, low-density neutrophil; MDSC, myeloid-derived suppressor cell; PD-1/PD-L1, programmed cell death 1 / ligand 1; TIM-3, T-cell immunoglobulin and mucin-domain containing-3; CTLA-4, cytotoxic T-lymphocyte–associated protein 4; ROS, reactive oxygen species; NET, neutrophil extracellular trap.
