# Supplementary material for: Inflammation-driven immune reprogramming in sepsis: from cytokine storm to immunoparalysis
Source: Front Immunol. 2026 Jul 9;17:1887033. doi: 10.3389/fimmu.2026.1887033 (PMC13391346; doi:10.3389/fimmu.2026.1887033)
Supplement: Supplementary file 4 [file Table3.docx]

**Supplementary Table S3. Immune Endotypes Across Cohorts**

| **Endotype (label)** | **Dominant modules (inflam/coag/adaptive etc.)** | **Key gene / cell patterns (examples)** | **Kinetic tendency** | **Outcome association (typical)** | **Simplified bedside proxy (feasible)** |
| --- | --- | --- | --- | --- | --- |
| Hyperinflammatory / Thrombo-inflammatory | IL-1/TNF/IL-6 axes, endothelial activation, NET–platelet crosstalk | ↑IL1B/TNF/IL6; neutrophil activation; NETosis signals; endothelial injury features | Early peak; may persist in “storm-dominant” trajectory | Early organ failure risk; shock/ARDS tendency | Ferritin↑, CRP/PCT trend; lactate kinetics; platelet consumption pattern |
| Coagulopathic / Endothelial-leak | Coagulation–fibrinolysis imbalance, microvascular dysfunction | Platelet–coagulation signatures; immunothrombosis pattern | Early–mid course; often overlaps with inflammation | Higher DIC/microthrombi burden; organ perfusion failure | Platelet↓ + D-dimer↑/INR↑; bedside DIC score components |
| Innate tolerant / Immunoparalysis-leaning | Antigen-presentation suppression, IL-10/TGF-β counter-regulation | ↓MHC-II/HLA-DR module, HLA-DR^low monocytes; blunted LPS-response | Can appear very early; may consolidate over days | Secondary infection / late mortality risk | mHLA-DR↓; “ex vivo LPS TNF” (if available) |
| Adaptive depleted / Exhaustion-leaning | Lymphocyte loss + checkpoint dominance | ALC↓, apoptosis signatures; ↑PD-1/PD-L1/CTLA-4/TIM-3 patterns; dysfunctional T/NK clusters | Often emerges within days; slow recovery | Viral reactivation; nosocomial infection; chronic critical illness risk | ALC↓ trend; (optional) flow checkpoint panel if available |
| Emergency myelopoiesis / Immature myeloid–MDSC-like | Stress hematopoiesis, immature granulocytes, suppressive myeloid programs | Immature neutrophils/LDNs↑; MDSC-like suppression markers; arginine-depletion logic | Builds over days; prominent in extreme trajectories | “Quantity–function paradox”, persistent organ injury + infection vulnerability | Immature granulocyte fraction↑; NLR trend; persistent leukocytosis with dysfunction suspicion |
| Mixed / Intermediate (parallel activation–suppression) | Concurrent inflam + tolerance/exhaustion modules | Co-expression of inflammatory cytokine modules + low-response markers | State-switching common; trajectory-dependent | Explains heterogeneity + trial neutrality | Composite: ferritin + mHLA-DR + ALC (serial) |

**Note:**

Modules and markers are shown as archetypal examples; specific gene sets and cluster labels vary by platform and cohort. Kinetic tendency should be interpreted with time-stamped sampling and treatment context.

**Abbreviations:** ALC, absolute lymphocyte count; mHLA-DR, monocyte HLA-DR; MDSC, myeloid-derived suppressor cell; LDN, low-density neutrophil; NET, neutrophil extracellular trap.
